# A Soft Reconfigurable Circulator Enabled by Magnetic Liquid Metal Droplet for Multifunctional Control of Soft Robots

**DOI:** 10.1002/advs.202300935

**Published:** 2023-06-13

**Authors:** Yi Xu, Jiaqi Zhu, Han Chen, Haochen Yong, Zhigang Wu

**Affiliations:** ^1^ Soft Intelligence Lab State Key Laboratory of Digital Manufacturing Equipment and Technology Huazhong University of Science and Technology Wuhan 430074 China

**Keywords:** liquid metal, logic computing, programming, reconfigurable modules, soft robotic control

## Abstract

Integrated control circuits with multiple computation functions are essential for soft robots to achieve diverse complex real tasks. However, designing compliant yet simple circuits to embed multiple computation functions in soft electronic systems above the centimeter scale is still a tough challenge. Herein, utilizing smooth cyclic motions of magnetic liquid metal droplets (MLMD) in specially designed and surface‐modified circulating channels, a soft reconfigurable circulator (SRC) consisting of three simple and reconfigurable basic modules is described. Through these modules, MLMD can utilize their conductivity and extreme deformation capabilities to transfer their simple cyclic motions as input signals to programmable electrical output signals carrying computing information. The obtained SRCs make it possible for soft robots to perform complex computing tasks, such as logic, programming, and self‐adaptive control (a combination of programming and feedback control). Following, a digital logic‐based grasping function diagnosis, a locomotion reprogrammable soft car, and a self‐adaptive control‐based soft sorting gripper are demonstrated to verify SRCs’ capabilities. The unique attributes of MLMD allow complex computations based on simple configurations and inputs, which provide new ways to enhance soft robots' computing capabilities.

## Graphical abstract

Integrated control circuits with multiple computation functions are essential for soft robots to achieve diverse complex real tasks. Utilizing simple and cyclic smooth motions of magnetic liquid metal droplets in specially designed and surface‐modified circulating channels, a soft reconfigurable circulator is proposed to achieve complex and multiple computing functions, such as logic, programing, and self‐adaptive control with simple control strategies.

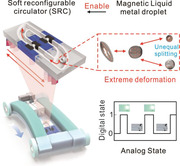

## 1 Introduction

Recently, soft robots have demonstrated various promising ways for simplifying interactions with complex environments and humans, applying their flexible materials and compliant structures to easily perform various tasks that are difficult for rigid counterparts, such as manipulating fragile objects and navigating through debris.^[^ ^1^ ^]^ Various principles have been presented for the actuation of soft robots, including pneumatic,^[^ ^2^, ^3^ ^]^ hydraulic,^[^ ^4^, ^5^ ^]^ tendons,^[^ ^6^, ^7^ ^]^ and smart materials.^[^ ^8^, ^9^, ^10^ ^]^ Functional behaviors, such as grasping,^[^ ^11^, ^12^ ^]^ swimming,^[^ ^13^ ^]^ and jumping,^[^ ^14^ ^]^ can be easily realized by the single stimulus of these actuation principles, enabling soft robots to be used in various areas, such as minimally invasive surgery,^[^ ^15^, ^16^ ^]^ industry,^[^ ^11^ ^]^ and field exploration.^[^ ^14^ ^]^ However, as the application scope of soft robots gradually expands, compliant materials and structures combined with a single stimulus are difficult to handle many circumstances' needs of real tasks. More complex sequence, logic, or even self‐adaptive control of multiple actuators are highly demanded to further enhance the functionality of soft robot systems, where all these control strategies need to be implemented by embedding computation in the systems.

For most soft machines above the centimeter scale, the embedded computation means integrating a network of sensors, corresponding signal and voltage conversion modules, and hard electronic microcontrollers (Figure S1, Supporting Information).^[^ ^17^, ^18^, ^19^, ^20^, ^21^, ^22^, ^23^ ^]^ However, a corresponding complex control system consisting of these components that enables complicated and coordinated actuations will undoubtedly limit the structural simplicity of a soft robot system. Up to now, the main efforts to overcome such limitations are concentrated on the development of soft fluidic control circuits. Like electric currents, fluidic pressure can act as either/both an actuation power or/and an information carrier, enabling new possibilities for constructing fully fluid‐based soft robots with computing functions.^[^ ^24^, ^25^ ^]^ However, pneumatically based computing systems deprive many advantages of electronic‐based computing systems, for example, compatible with versatile electronic functional components, and various actuation principles including shape memory alloys, dielectric elastomer actuation (DEA), electro adhesion, etc. Thus, by utilizing the conductivity of some specific fluids, researchers have tried to convert the controlled fluid movement into electrical signals within simple soft fluidic circuits and demonstrated some interesting soft machines with various computing functions.^[^ ^26^, ^27^, ^28^ ^]^ Although basic computation functions, such as feedback, programming, and logic control, have been demonstrated in current works, realizing combined applications of multiple computing functions within a simple structural framework is still a tough challenge for soft robots. This is mainly owing to the lack of effective techniques (e.g., regulating the internal and external forces) to manipulate the deformation and smooth movement of conductive fluids in micro/fluidic channels since those fluidic channel networks are typically designed with a well‐defined protocol under sealing conditions. Therefore, all these factors severely limit the potential of some unique properties of fluids in enabling complex computations and restrict possible structures and function designs of current relevant soft controllers (most are based on tubular structures).

Liquid metal (LM) is conductive and highly compliant with outstanding deformability in the open air. Thus, manipulation of the deformation and movement of such conductive fluids not only requires no sealing condition but also enables complex computations of control circuits with various new design possibilities.^[^ ^29^, ^30^ ^]^ Moreover, remote control of its deformation and movement can be realized by dispersing magnetic particles into an LM droplet,^[^ ^31^, ^32^ ^]^ which further enriches the manipulation ways of LM droplets. Consequently, such magnetic LM droplets or the so‐called MLMDs, not only inherit the LM's characteristics,^[^ ^33^, ^34^, ^35^, ^36^, ^37^ ^]^ but also can be treated as a deformable host of magnetic particles.

**Figure 1:**
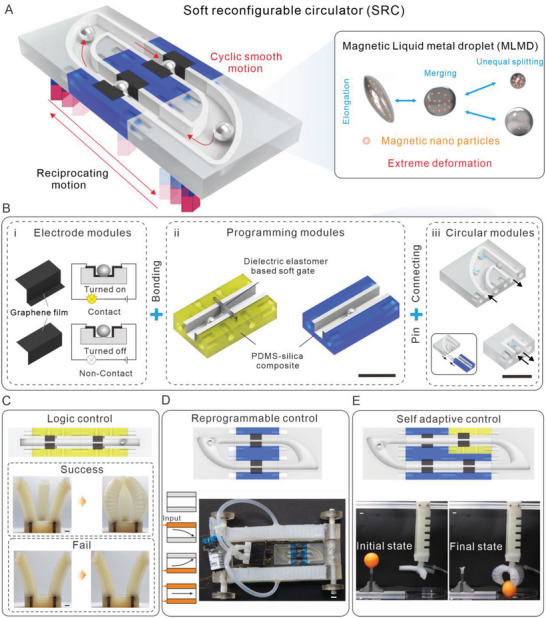
Concept of MLMD enabled multifunctional SRC. A) Schematic of MLMD enabled SRC. MLMD maps a reciprocating motion of a magnetic signal to a cyclic electrical output signal. Both logic computing and programing can be achieved (right). B) Three fundamental modules. i) The electrode modules are used to attach on the programing and circular modules to trigger the electrical conducting behavior via MLMD. Two typical electrodes are designed to achieve selective conduction based on liquid metal size. The electrode above (non‐selective electrode) can be conducted by all MLMDs, while the electrode below (selective electrode) can only be conducted by large‐sized MLMD. ii) Programing modules are applied to achieve sequential predesigned conducting behavior. Noting that DEA‐based soft gates are always integrated with yellow modules to trigger extreme deformation behavior that results in information storage and control mode switching. iii) Circular modules are always connected with programing modules to guide the periodic motion of MLMD. Two typical circular modules are designed to implement one‐way and reciprocating cycles of MLMD, respectively. C) Logic computing‐based grasping function diagnosis. Implementation of two “And” logic gates configuration enables simple decision‐making (grasping or not). D) Integrated reprogrammable control of a soft car. Five different mode control can be achieved for diverse gaits based on the structural design of soft materials. E) Robot that uses soft materials with structural compliance to achieve gripping and sorting behavior when interacting with an unconstructed environment. Scale bars, 1 cm.

Utilizing their high conductivity and extreme deformability of MLMDs, herein, we introduce a soft reconfigurable circulator (SRC) that maps a cyclic motion of MLMD as an input signal to complex electrical output signals via soft electrodes embedded into a soft channel, **Figure** **1**A, where a simple, intermittently reversible magnetic field is acted as the power of MLMD cyclic motions. The SRCs consist of three basic reconfigurable modules that are designed to realize the energy conversion, extreme deformation, and cyclic motion of MLMD, respectively, Figure 1B. Therefore, a library of soft modules can be created and reconfigured to form a multifunctional controller toward system‐level completeness. Compared with other existing soft fluidic control circuits, our SRCs have better overall performance in terms of input simplicity, functional diversity, structural reusability, and compatibility (detailed comparisons can be found in Table S1, Supporting Information). As a proof of concept, the SRCs are demonstrated to achieve diverse complex computation tasks for soft matter systems, for example, decision‐making, reprogramming, and even environmental adaptation, Figure 1C–E.

## 2 Results and Discussion

### 2.1 Concept of MLMD Enabled Multifunctional SRC

For programs running in rigid microcontrollers, a loop is a key structure that enables the initialization and continuous operation of computation behavior. Similarly, in soft alternatives, we can also construct circular work mechanisms to promote the continuous operation of various computing functions. Considering this issue, we physically implement the loop structure of programs as a simple cyclic motion of MLMD in UV‐laser micromachined channels, and thus propose the concept of the circulator. Further, the main body of the circulator can be physically modularized like program instructions to achieve convenient reprogramming, where the unique stimulus‐response of MLMD can be used to enable various complex computations. To develop a reconfigurable and simple circulator capable of achieving multiple computations, three fundamental modules are designed with flexible materials, which are electrode modules, programming modules, and circular modules, respectively, Figure 1B. The electrodes can be easily attached to the soft programming and circular modules (Figure S2, Supporting Information), which can be easily connected with soft connectors (Figure S3, Supporting Information).

**Figure 2:**
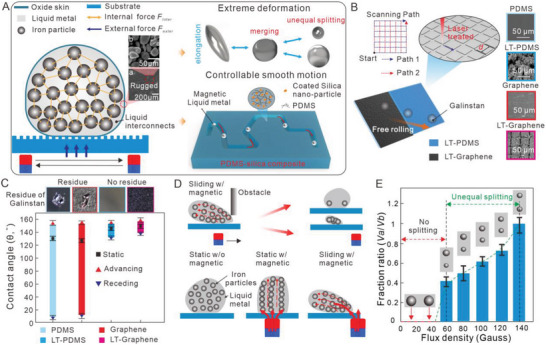
Non‐wetting characteristics and extreme deformation of MLMD for SRC. A) Schematic of internal and external forces acting on MLMD for extreme deformation and controllable smooth motion. B) Schematic of the fabrication process for the non‐adhesive UV laser micro‐textured substrates to an MLMD, left panel. Scanning electron microscopy of modified and non‐modified substrates (scale bar: 50 µm), right panel. LT means laser‐textured surface. C) Advancing, receding, and static contact angle of the MLMD on different surfaces, lower inset. Residues remaining on the retracting MLMD surface, upper inset. D) Schematic of iron particle rearrangement under the magnetic and shear force, where w/ means with magnetic, and w/o means without magnetic. E) The volume fraction of two split MLMD as a function of the magnetic flux density.

As a cyclic motion‐based circulator is aiming to mimic the loop in programs physically, manipulating the mechanical response of MLMD, such as smooth motions and extreme deformations, is very important. The mechanical response of MLMD can be achieved by regulating the internal and external forces (e.g., magnetic force, friction force, and shear force) applied on it, **Figure** **2**A. The smooth motion of MLMD is enabled by laser‐based processing of soft module surfaces, which can enhance the non‐wettability of MLMD (more details in Section 2.2), while the control of extreme deformation of MLMD is realized by regulating the shear force and magnetic force with opposite directions applied on the MLMD (more details in Section 2.3). Simultaneously, we also study the mechanism and characterization of controlled motion of MLMD on SRC's computing performance in Section 2.4, investigate digital/analog output and electrical performance of SRC in Section 2.5, and demonstrate the capabilities of SRCs by utilizing them to enable the multifunctional control of soft systems in Section 2.6.

### 2.2 Non‐Wetting Characteristics of MLMD

As revealed by the previous study, gallium‐based liquid metals have significant stickiness at the interface between liquid metal and solid surface.^[^ ^38^ ^]^ To overcome the significant stickiness problem in achieving controllable smooth motion of MLMD outside a liquid environment, a one‐step, cost‐effective strategy with laser micromachining is introduced, Figure 2B, left panel. The previous study shows that silica nanoparticles coated with polysiloxanes can enhance the surface repellency to LM.^[^ ^39^ ^]^ Therefore, the coated silica nanoparticles were mixed into silicone elastomer, and then the cured surface was patterned with an UV laser, Figure S4 (Supporting Information). There, both micro/nanostructures and silica nanoparticles exposure can be controlled, Figure S5 (Supporting Information).^[^ ^40^, ^41^ ^]^ Same procedure is applied to the graphene film surface, Figure S6 (Supporting Information). The scanning electron microscopy (SEM) images show micro concaves alignment on both laser textured surfaces, thus enhancing the non‐wettability of MLMD, Figure 2B, right panel, and Movie S1 (Supporting Information). The designed shape of the micromachined channel is a parallelogram as that not only facilitates UV laser scanning with uniform light absorbance to solve the sticky problems of MLMD but also enhances the compact design of SRC with better assembling and connection of modules and channels. However, other shapes, for example, reniform or circular ones may provide less resistance for running the MLMD. Thus, a trade‐off of various factors should be carefully considered during the design, depending on the practical scenarios or specified targets. The preparation procedure of MLMD is shown in Figure S7 (Supporting Information). By controlling different volume fractions of iron particles in Galinstan (GaInSn), the magnetohydrodynamic (MHD) phenomena can be observed by the interaction of the magnetic field with fluid flows.^[^ ^42^ ^]^ Therefore, this smart fluid can respond immediately to an applied magnetic field and features reversible change from liquid to semi‐solid or solid state,^[^ ^43^ ^]^ Figure S8 (Supporting Information).

To further demonstrate the effectiveness of such a strategy, the non‐wetting characteristics of MLMDs with/without UV laser micromachining are investigated. It is well known that yield stress will prevent the LM from flowing to obtain a minimal surface area that enhances the wetting behavior of MLMDs.^[^ ^44^ ^]^ Moreover, the formation of an oxide skin on LM surface causes significant stickiness problems to most substrates that seriously affect the available applications of LM.^[^ ^45^ ^]^ Consequently, without UV laser texturing, the MLMD droplets exhibit a relatively high wettability on PDMS and graphene surfaces, respectively, Figure 2C, lower inset. Additionally, residues can be observed remaining on the retracting MLMD surface. By contrast, no residues are observed on the laser‐textured PDMS and graphene film surface, Figure 2C, upper inset. This is due to the fact that, according to the wetting properties,^[^ ^46^ ^]^ the adhesive force between a liquid and a solid is stronger than the cohesive forces within the MLMDs. Thus, to alter the adhesive force, laser‐induced micro/nanostructures can be formed on these elastomer surfaces to endow them with lower surface energy. Moreover, the exposure of coated silica nanoparticles with low‐surface energy (polysiloxanes) can enhance the repellency to MLMDs with a large static contact angle.^[^ ^47^ ^]^ Therefore, controllable smooth motion of MLMDs outside a liquid environment can be achieved with modified surfaces in confined channels, Movie S1 (Supporting Information), and thus it enables the smooth cyclic motion of MLMDs under the control of simple reciprocating magnetic flux, Figure S9 (Supporting Information).

### 2.3 Mechanism and Characterization of Extreme Deformation of MLMDs

To realize the function of program initialization and switching of SRC, unique capabilities in terms of extreme deformation of MLMD are investigated. Here, the controllable shape variation, splitting, and merging behaviors of MLMD subjected to internal and external forces are observed, Movie S2 (Supporting Information). According to previous studies,^[^ ^48^ ^]^ iron particles alloyed with liquid metal change from random orientation to chain or column structures under magnetic flux. Therefore, when the magnetic orientation changes, the structure of the chain or column will be altered accordingly to configure new shapes of MLMD, Figure 2D, lower panel. As in Figure S10 (Supporting Information), under the combined effect of extreme shear forces and magnetic forces, the oxide skin of MLMD will break and the inner metal will flow out from the LM as a low‐viscosity liquid, owing to that the surface stress of the oxide skin exceeds the critical point of 0.5–0.6 NM^−1^.^[^ ^49^ ^]^ Of course, such a break and regeneration of the oxide skin may affect the electrical conductivity of MLMDs. However, in our case, it is not a big issue since a relatively heavily oxidized LM^[^ ^48^ ^]^ was not observed. An interesting phenomenon of iron particle distribution for the split MLMD can be observed by energy dispersive X‐ray (EDX) characterization, Figure 2D, upper panel. The EDX results show that almost no iron particle (0.43%) could be detected from one splitting MLMD, Figure S11 (Supporting Information), right panel, while the other splitting MLMD droplet has significantly higher iron particle content (1.63%), Figure S11 (Supporting Information), left panel. More importantly, for the splitting behavior, by adjusting the relative strength of magnetic flux, we can control the volume ratio between *V*_a_ (the one with almost no iron particle) and *V*_b_ (the one with higher iron particle content), of the resulting two sub‐MLMD, as in Figure 2E. When the magnetic flux is less than 60 Gauss, the shear stress induced by the magnetic flux is not strong enough to overcome the surface tension to trigger any splitting (the “no‐splitting” phase). However, when the magnetic flux is larger than 60 Gauss, split into small droplets with various splitting volume ratios can be achieved according to the volume fraction ratio *V*_a_ */V*_b_. Meanwhile, the spitting MLMDs will merge together and move as the original one, since alloying dynamic process between LMs always happens at room temperature.^[^ ^50^ ^]^ Such a splitting behavior (Figure S10, Supporting Information) and unequal iron particle distribution (Figure S11, Supporting Information) is the key factor for the multifunctional control design in logic and adaptive control, which can be clearly seen in the later section, for example, that in **Figures** **4**a and **6**.

Subject to internal and external forces, the extreme deformation of MLMD is demonstrated to be well controlled. Thus, program initialization and switching can be implemented. For instance, based on the splitting phenomenon, a DEA‐based soft gate integrated with soft programming modules (yellow) can be designed to receive external signals and decide whether to split or block the MLMD, which may lead to the program switching of SRC, Figures S12 and S13 (Supporting Information). Also, after the program is computed and executed, the merging phenomenon of MLMD will trigger the initialization of all the programming. Therefore, leveraging simple cyclic motion and extreme deformation behavior of oxidized MLMD can realize a new computational mechanism with programmable capability. As a consequence, a number of design possibilities for SRC to enhance computational capabilities can be explored.

### 2.4 Mechanism and Characterization of Controlled Motion of MLMD

**Figure 3:**
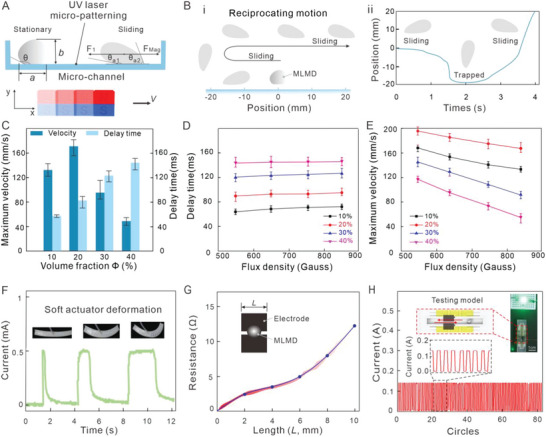
Mechanism and characterization of SRC computation. A) Schematic description of stationary and sliding MLMD (only force components in the *x*‐axis are depicted) under magnetic flux, where *F*_1_ and *F*_Mag_ are friction and magnetic force, respectively. *θ*_a1_ and *θ*_a2_ are defined as receding and advancing angles, respectively. B) Sliding of an MLMD in the presence of a moving permanent magnet (*B* = 120 mT, *v* = 40 mm s^−1^). i) Evolution of the moving MLMD during reciprocating motion. ii) Displacement of the MLMD versus time. C) Maximum velocity and delay time of MLMD versus iron‐nano particles in Galinstan as a function of volume fraction Φ. D) Delay time of the MLMD versus flux density. E) Maximum velocity of MLMD versus flux density. F) Single computing unit acts as an amplifier or filter to control the deformation of the soft actuator. G) Electrical resistance of different lengths (*L*) of electrodes with MLMD. H) The stability of a single programming module is tested with PWM outputs.

The kinematics of the sliding motion and shape deformation of MLMD are highly related to the performance of SRC, for example, program computing time (conducting time with electrodes) and program running speed (moving speed in the channel). **Figure** **3**A presents the fundamental mechanism of MLMD's controlled motion from stationary to a sliding state under magnetic flux. The delay phenomenon can be observed under a high‐speed camera, Figure 3B and Figure S14 (Supporting Information). Time‐dependent images of MLMD from the middle to the left, and then right (reciprocating motion) are in Figure 3B(i). Figure 3B(ii) shows a clear trapping zone (no displacement) during the back‐and‐forth motion of the magnetic field. Referring to the contact angle changing behaviors of MLMD at the same period in Figure 3B(i), only the shape deformation of MLMD is observed without any movement. Hence, the phenomenon should be strongly relevant to the formation of the trapping zone as seen in Figure 3B(ii). Therefore, the shape deformation (contacting angle changes) is supposed to seriously influence the program's running speed during the circular motion, and we define the time in the trapping zone as the delay time.

To learn the performance of SRC with delay time and running speed, different volume fractions of MLMD and magnetic flux densities are investigated. Figure 3C shows that under the same magnetic density (850 Gauss), the minimum delay time is found at the lowest iron volume fraction while the maximum velocity is achieved at the 20% iron volume fraction. The results imply that there might be a competitive relationship between magnetic and friction force that will increase and decrease the velocity of MLMD. Moreover, the flux density applied on MLMD has little influence to the delay time, while the delay time increases with the volume fraction increment, Figure 3D. The phenomenon indicates that the internal magnetic force of iron particles during the ferromagnetic rearrangement process may be the main reason for the delay time. Figure 3E shows that the maximum velocity of MLMD decreases with the flux density increment. Such a phenomenon further verifies the competitive relationship between magnetic and friction forces. When the magnetic flux density in the vertical direction increases, the magnetic particles will gather more closely to the contacting surface. Consequently, the surface of MLMD and substrate will contact more tightly with a larger contact area. Thus, higher friction force acting on the MLMD will hinder the maximum velocity of MLMD in the horizontal direction. It hints that the maximum velocity is highly related to two factors: magnetic force and friction force acting on MLMD. Therefore, both the volume fraction Φ of MLMD and the applied magnetics flux density can be optimized to achieve higher computing efficiency (lower delay time and higher running speed).

### 2.5 Function Verification

From the perspective of system‐level design, the SRCs are expected to operate in either digital or analog modes. We demonstrate the working principle of a single programming module by using it to control a light‐emitting diode, Movie S2 (Supporting Information). The geometrical parameter *H* of MLMD, the electrode length *L*, and the gap between electrodes *d* are the key parameters that decide the “on” and “off” states and their duration of a programming module, Figure S15 (Supporting Information). In the analog mode, we demonstrate that under a constant velocity of MLMD, the magnitude of electrode length *L* can be used to regulate the bending angle of a soft bending actuator by controlling its actuation time, Figure 3F. While in digital mode, the MLMD conducting behavior can be defined as output “1”, else “0”, Figure S16 (Supporting Information). Therefore, SRC can be composed of several programming modules to perform digital computing. During our investigations, we found that the geometrical parameters of MLMD are related to the magnetic flux density as the shape deformability of MLMD under different magnetic flux densities is observed, Figure S17 (Supporting Information). Also, two typical types of graphene film electrodes are designed to achieve selective conduction based on LM size, Figure S18 (Supporting Information). In addition, the required magnetic flux density can be generated in various ways, such as a remote magnetic generator, an electrically induced magnetic field, or a controlled moving permanent magnet.^[^ ^51^, ^52^ ^]^ In our experiments, the controllable magnet is manually moved periodically in a straight line at the bottom of the soft machines. Therefore, the MLMD can be guided by the generated magnetic field to move at a specified speed.

To assess the electrical performance of a single programming module, a number of prototypes with different *L* and fixed gap *d* are tested. The results show that the resistance will significantly increase as *L* increases, Figure 3G. Also, the resistance here (non‐liquid conductive environment) is three orders of magnitude less than that in the liquid conductive environment.^[^ ^19^ ^]^ Therefore, a number of soft functional materials that need to work under low resistance can be controlled with SRC. Moreover, the stability of electrical performance for the programming module is tested at a constant speed of 25 mm s^−1^, Figure 3H. The testing result shows that a cyclic output can be generated with the reciprocating motion of MLMD, Figure 3H, zoomed‐in window, and Figure S19 (Supporting Information). Therefore, the signals can be either amplified or filtered with predefined electrode length *L* and the gap between electrodes *d* as pulse‐width modulated (PWM) outputs. Further results show that the output current does not change significantly after 80 reciprocating cycles, which indicates that SRC has a good stability of electrical performance. However, these might still suffer from sticky problems under two conditions. One is that when running cycles many times, there might be the fatigue of the electrodes and the sticky phenomenon may be observed. The other is that when the output current is too high, the oxide thickness will grow fast since high enough electric field strength will facilitate oxide growth significantly.^[^ ^38^ ^]^ Gradually, the stability of SRC will be degraded by the increased adhesion between electrodes and MLMD. Further optimization will be necessary in future.

### 2.6 Multifunctional Control of Soft Robots with SRC

To demonstrate the multifunctional control with SRC in system‐level design, three typical SRC configurations are designed by applying the basic module libraries. Further, we use these configurations to enable three kinds of soft matter systems that can display versatile computation capabilities, such as decision‐making, reprogramming, and environment adaptation.

#### 2.6.1 Digital Logic Computing‐Based Grasping Function Diagnosis

**Figure 4:**
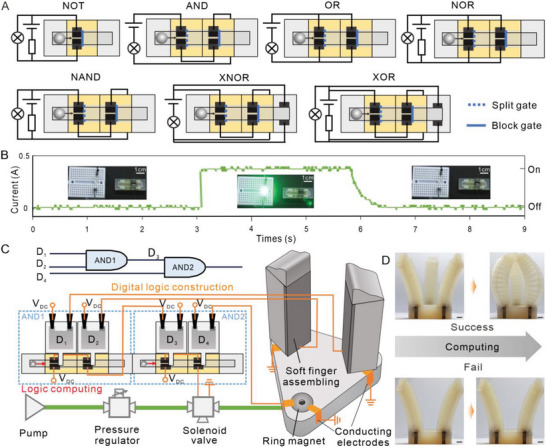
Digital logic control of SRC for decision making. A) Schematic of a full set of logic gates achieved by the connection of the electrodes with serial or parallel arrangements. B) A NOT gate is displayed for the basic working principle of logic computing. C) A logic circuit consisting of two AND logic gates exhibits both information storage and computing function for decision‐making. Assembling information of soft fingers is stored within the programming module (yellow) as digital logic construction, and the decision‐making behavior is decided by the digital computing process with MLMD. D) When the soft fingers are assembled in the correct position, the gripper will be actuated. Otherwise, the program will not be executed. Scale bars, 1 cm.

Yellow programming module, reciprocating circular module, and electrodes are used to construct the SRC configuration for digital logic computing. Figure 4A shows the schematic of seven possible sub‐configurations corresponding to a full set of logic gates: AND, OR, NOT, NAND, NOR, XNOR, XOR. The NOT gate only requires one yellow programming module with a block gate (the groove is the same height as the bottom of the channel, which can be used to control whether the MLMD is blocked or not). While other gates need two yellow programming modules with a split gate (the groove is higher than the bottom of the channel, which can be used to control whether the MLMD is split or not) and a block gate, respectively. The baffles in the block or split gates can be regarded as the input signals of the logic gates (if the input is “1”, there is a baffle in the groove; while if “0”, there is no baffle), while the expected truth table output is demonstrated with the LED lights up, else with LED lights off. In addition, it should be noted that the circular motion of MLMD here can be used to control the start and initialization of logic gates. The motion of MLMD from left to right can be regarded as a computing process, which may involve a splitting behavior, while the motion of MLMD from right to left can be regarded as program initialization, which may involve the merging of several MLMDs. Figure 4B shows the current variation during NOT gate computing, while the true value table of other gates and the final state of each output are demonstrated in Figures S20 and S21 (Supporting Information). Also, the logic computing of all these SRCs can be found in Movie S3 (Supporting Information).

By assembling multiple SRC‐based logic gates in series, multi‐level logic gates can be implemented to enable more complex logic computations, such as decision‐making with multiple inputs. As a proof of concept, we assemble two SRC‐based AND gates in series to form a simple secondary logic gate and further achieve function diagnosis of a three‐finger soft gripper modular, Figure 4C and Figure S22 (Supporting Information). To automatically input external signals to SRC, DEA‐based soft gates are integrated with yellow programming modules to control the splitting or blocking of MLMDs. To realize information transmission between two AND gates, the output signal of the first AND gate will be used to control the corresponding DEA‐based soft gate of the second AND gate as an input signal, Figure 4C. The pneumatic modular is composed of a soft base and three soft fingers. Soft fingers can be connected and automatically aligned with the base through the embedded ring magnets, Figure S23 (Supporting Information). When the soft fingers are successfully assembled and oriented correctly (necessary conditions for the gripper to work properly), the copper foils on them will contact with that on the soft base to conduct the corresponding diagnostic circuits, which will control the corresponding DEA‐based soft gates to descend and insert their baffles into the grooves as the input “1”, Figure 4C and Movie S4 (Supporting Information). The secondary logic gate consisting of two AND gates means that all three fingers must be connected successfully (all three inputs are “1”) for the final output signal to be “1”. The final output signal will be used to determine whether the pneumatic gripper should be actuated for grasping or not. Therefore, only if all the fingers are successfully assembled and oriented correctly, the soft gripper will be actuated to grasp when the computing process is finished, Figure 4D. On the contrary, if any of the soft fingers are not correctly assembled, the action will not be executed, Movie S5 (Supporting Information).

SRC‐based logic gates allow soft systems to achieve logic computing functions in a compatible and simple way, and more complex logic computations can be implemented by combining several SRCs together. Moreover, a key feature of our SRC‐based logic gates is that the computing and input process can be carried out separately. This means that we can input external signals through DEA‐based soft gates first, and then choose to perform computation later when needed. This feature allows our logic gates to have register‐like or latch‐like properties, thus enabling more flexible computations.

#### 2.6.2 Locomotion Reprogrammable Soft Car

**Figure 5:**
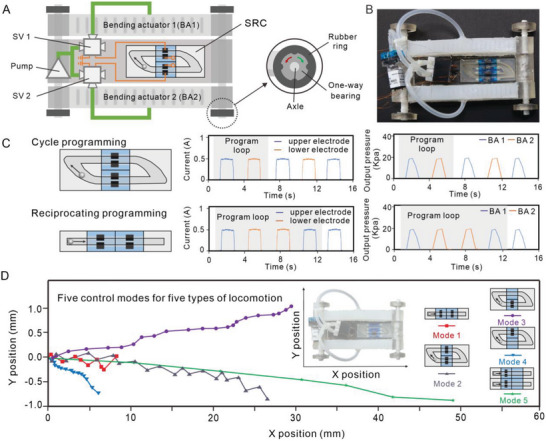
Integrated SRC for reprogrammable control. A) Top view of the SRC embedded into the soft matter system with all the electronic components assembling together in a compact design, where SV means solenoid valves. B) Soft car moves by exploiting the contraction force (bending actuators BA1 and BA2) to drive the front wheel and rear wheel moving forward. C) Reconfiguration of fundamental modules to form different programing patterns with defined actuation periods and orders. Two typical program running modes, left panel. Output for an applied current of 0.5 A, with a blue line for the upper electrode, and an orange line for the lower electrode, middle panel. Output pressure for two bending actuators, right panel. D) Different locomotion behaviors are controlled by the reconfiguration of fundamental modules under five different control modes.

Following, we demonstrate the SRC configuration for programming by integrating its sub‐configurations into the body of a soft car endowed with diverse locomotion modes. The soft car moves by exploiting the contraction of two bending actuators (BA1 and BA2) connected with the front and rear one‐way wheels. Its detailed locomotion principles can be seen in Figure S24 (Supporting Information). **Figure** **5**A,B shows the top view of the SRC embedded into the car with all the electronic components assembled together in a compact design. Blue programming module (without groove), circular modules, and non‐selective electrodes are used to construct the SRC configuration for programming. As in Figure 5C, left panel, the SRC can realize the one‐way and reciprocating motion of MLMD with two kinds of circular modules, respectively. As a consequence, both cycle and reciprocating programs can be achieved in different operation modes under the same magnetic field. Noting that the two actuators of the car have two different cycle actuation periods, for example, alternate actuation of BA1 and BA2 can be defined as one cycle for cycle programming, while two inverse actuation of BA1 and BA2 can be defined as one cycle for reciprocating programming, Figure 5C, right panel. Further, by freely reassembling basic modules, five different gaits of the car can be generated, for example, turning, serpentine, and forward moving, Figure 5D and Movie S6 (Supporting Information). In addition, due to the imperfect molding and assembling process of soft components, an asymmetrical expansion of left bending actuator (BA1) and right bending actuator (BA2) is found always unequal in our test (Figure 5A). Thus, there is a downward shift observed in modes 2 and 5, even though modes 2 and 5 are symmetrical and mode 4 has a quite dense set of value points. In the future, an improved fabrication will be sought to minimize such an issue.

By adjusting the electrode length *L*, we can regulate the running time of the single step, which directly determines the bending degree of the two actuators. As in Figure 5D, the maximum forward speed of the soft car can be achieved at control mode 5 (forward moving gait) with 0.3 mm s^−1^ and the maximum turning angle can reach up to 0.4° s^−1^ at control mode 4 (right turning gait). Although longer *L* will enable larger bending force for higher forward or turning speed of the car, the soft bending actuators will be broken when the internal pressure exceeds the limit of the soft structural material.

Preliminary, we show the capability of SRC configuration for programming through a soft car demonstration. More complex programs can be easily constructed and reprogrammed by introducing more blue programming modules in this way. Also, SRCs can be integrated into soft structures without worrying about weakening the overall compliance of soft machines. This is because their soft‐material‐based bodies with the compliance and semi‐liquid properties of MLMDs enable the SRCs to deform compliantly and maintain stable working performance.

#### 2.6.3 Self‐Adaptive Control of a Soft Sorting Gripper

**Figure 6:**
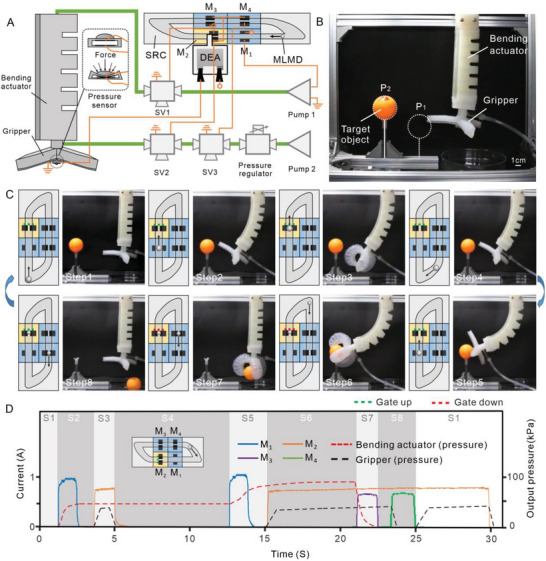
SRC enabled self‐adaptive control. A) The sequential bending and gripping actions of the soft robot are controlled by air pressure through solenoid valves (SV) and pumps. The states of SV(1–3) are controlled by contacting behavior between MLMD and electrodes of SRC. B) The soft robot will repeat the bending and gripping action for each circle trying to catch the target object. C) Sequential steps of the automatic sorting behavior are demonstrated with adaptive control. The gripper can interact with an unstructured environment to trigger the program switching. When the targeted object is locked, the gripper will automatically grasp it to the target place as a sorting robot. Then, the program initialization of SRC will be activated to start new sorting behavior. D) Actuating current and air pressure required in sequence for the self‐adaptive control. In steps S1–S5, the gripper tries to grasp the targeted object and the program 1 are executed with bending and grasping action. Once the gripper catches the object (S6), the program 2 is triggered. In steps S7–S8, the soft gripper will relax the soft arm and gripper in sequence to put the object in the box as the sorting robots. Then, the splitting MLMD will merge together to initiate the program to start a new circle (Step 1).

To further demonstrate SRC's functionality, we present an SRC configuration that combines programming and feedback control to achieve a self‐adaptive control (adaptive automatic switching of programs) and use this configuration to control and enable a soft sorting gripper that can find an object placed at any point of its bending trajectory and grab it into the collection box directly below, **Figure** **6**A,B. Programming modules, the one‐way circular module, and graphene film electrodes are used to construct the SRC configuration for self‐adaptive control. A DEA‐based soft gate is integrated with the yellow programming module with a splitting gate to accept external signals and control the splitting behavior of MLMD. The splitting and merging of MLMD will lead to a switching of the program. Especially, as mentioned earlier, the unequal distribution of iron particles during the splitting process causes different motions (moving or staying still) of MLMD. Therefore, the predefined programs can be selectively executed.

As in Figure 6A and Figure S25 (Supporting Information), the soft sorting gripper consists of a bending actuator and an end gripper. A simple flexible pressure sensor is attached to the center of the gripper and sends object contacting signals to the DEA‐based soft gate to control its rise and fall, Figure S26 (Supporting Information). Two sets of programs are pre‐designed within the SRC. One set of programs is to control the bending and gripping action, and the other is to control the relaxation of the soft bending actuator and the gripper. As shown in Figure 6C,D and Movie S7 (Supporting Information), once the MLMD starts to do a circular movement, the soft sorting gripper will repeat the bending and gripping action in each cycle, trying to catch the target object. As the gripper successfully grasps the targeted object, the flexible sensor will be pressed, actuating the DEA‐based gate to descend. Therefore, the MLMD will be split into two due to the shear force, and the second set of programs can be activated. Noting that the yellow module can not only store the information to trigger splitting behavior but also maintain the initial program and switch to another, Figure S27 (Supporting Information). As a consequence, the target object will be finally placed in the collection box directly below the gripper. After the gripper put down the object, the flexible sensor will return, and the DEA‐based gate will rise, allowing the two split MLMDs to merge and initialize the SRC (switch to the first set of the program again). The MLMD is doing a simple circular motion throughout the gripper's entire sorting process including finding, grasping, and putting down the item without any pause.

This demonstration shows that with a simple SRC, a sophisticated self‐adaptive control program can be implemented through the cyclic movement of MLMD. Such an SRC configuration for self‐adaptive control can be easily ported to other soft systems to provide a compliant and simple way to enhance their interaction capabilities with unstructured environments. For example, we can use our SRC to help soft mobile robots better adapt to different terrain by achieving adaptive switching of their motion modes. In addition, although this demonstration only shows the capability of SRC to switch between two sets of programs, it is possible to switch between more sets of programs by introducing more yellow programming modules in SRC, which is also one of our future works.

## 3 Discussion

In this work, we present a soft reconfigurable circulator, which provides a doable way for soft machines to achieve complex and multiple computation functions, such as logic, programming, and self‐adaptive control, with a simple structural framework. With the help of three simple and reconfigurable modules of SRC, MLMD can utilize its conductivity and extreme deformation capabilities to transfer its simple circulation movements (input signal) into complicated electrical output signals carrying computation results, and therefore enable soft robots to perform multifunctional computing tasks. Although conventional rigid circuit boards or flexible circuits with miniature rigid electronic components can do a similar job in a smaller form, some of our SRC's unique characteristics, such as softness and reconfigurability, can also offer some irreplaceable advantages to soft systems. For instance, our SRC might provide stiffness‐matched or even homogeneous interfaces and structures for possible integration when poor interfacial stability happens between rigid and soft structures.^[^ ^53^ ^]^ Moreover, our SRC can quickly switch computing functions and rewrite programs by quickly reassembling various modules, which can facilitate its application in diverse scenarios and tasks. By contrast, for traditional rigid electronic components, especially small‐sized ones, reprogramming might be complicated.^[^ ^17^ ^]^ Finally, although here only three typical configurations were demonstrated assembling by basic modules, the space for constructing new SRC architectures for embedding computing functions in soft systems is far beyond those presented.

To combine MLMD with modular SRC, first, we introduce a one‐step, cost‐effective strategy applying UV‐laser micromachining to overcome the stickiness problem of MLMD outside the liquid environment, and therefore enable the smooth motion of MLMD in the channel of SRC. Second, by regulating the internal and external forces of MLMD, we develop a method to control the extreme deformation of MLMD (splitting and merging) with physical baffle structures. Based on this method, we can transport external signals to MLMDs allowing them to implement functions such as logic computation and program switching. The results show that it is promising to introduce MLMD as a computing medium to enhance the computation capabilities of soft systems. Its unique properties, such as extreme deformation, can provide some interesting and novel ways to process information and may easily achieve some complex computations. In addition, other properties of MLMD or even unique properties belonging to alternative conductive liquids, such as surface shock waves and liquid natural penetration, could also be used to help soft machines process information and enhance their computing capabilities.

In our experiments, the MLMD's driving magnetic fields were generated by manually controlling a small permanent magnet to move periodically in a straight line at the bottom of the soft machines, Figure S28 (Supporting Information). The SRCs can help convert such simple magnetic control signals into complex electrical output signals through a relatively compact structure. Such an approach allows us to easily control the soft machines wirelessly to perform complex operations without introducing rigid signal‐receiving modules (such as a Bluetooth module) into soft systems. Therefore, the soft systems can ensure high softness yet stability to work in some demanding scenarios, such as rugged and narrow pipelines. In addition, as the widely commercially available reed switch can be combined with a magnet to enable non‐contact electrical switching for some devices, our SRCs can also be integrated with a soft magnet slide switch to enable manual switching for soft systems, as in Figure S29 (Supporting Information). Such a soft switch, enabled by SRCs, can synchronously complete function diagnosis (as that in Figure 4) or multi‐step pre‐startup (by using SRCs similar to those in Figure 5) of the soft systems during the sliding on the process and synchronously complete the initialization and multi‐step shutdown process (circular programming in Figure 5 allows the shutdown program to be different from the pre‐startup program) during the sliding off process. Thus, a convenient human‐soft robot interaction can be realized without significantly affecting the compliance and stability of the soft robot systems.

When it is necessary to build fully onboard SRC systems without external input magnetic signals, one feasible way is to introduce two complementarily activated soft electromagnets on both sides of the SRCs to drive MLMD's cyclic motion. As in Movie S8 (Supporting Information), by using two commercially available rigid electromagnets, MLMD can achieve smooth cyclic motion in the SRC and alternately conduct electrodes. By replacing the iron core with a soft silicone core doped with iron powder and replacing the outer coil with soft liquid metal wires, we can assemble a fully soft electromagnet that fits well into soft structures, as in Figure S30 (Supporting Information), which has been well proved by numerous works on soft electromagnets previously.^[^ ^54^, ^55^, ^56^, ^57^ ^]^ To further demonstrate the feasibility of this idea, we fabricated two soft iron cores and wrapped soft copper coils around them (which will be further replaced with softer liquid metal wires in the future) to drive the MLMD. As in Movie S9 (Supporting Information), the MLMD can achieve smooth motion within the channel of SRC and conduct electrodes under the actuation of the two soft electromagnets. In the future, we will further optimize the structure and size of the soft electromagnets to enable the compact and soft integration of SRC systems.

Another feasible way, which is also the next step we are working on, is to build an SRC‐based oscillator using the MLMD's own inertial forces or gravity to achieve continuous self‐cycling of the soft systems. For instance, as in Movie S10 (Supporting Information), the micromachined SRC channel allows MLMD to move smoothly within it under gravity and alternately conduct electrodes located at both ends of the channel. By integrating such an SRC into a screen disk with soft actuators mounted at both ends and using the SRC's two electrodes to drive the contraction of contralateral soft actuators, respectively, we can construct a self‐cycling SRC‐based screening oscillator under gravity, Figures S31 and S32 (Supporting Information). Equipped with an SRC, such a screening oscillator can automatically screen large and small spheres without additional input signals. We can manually start/stop the screening oscillator by using a small magnet to pull the MLMD to one end of the channel to conduct the electrode or hold the MLMD's position while cycling to prevent it from rolling to another end. Based on similar principles, it is also possible to construct an SRC‐integrated quadruped soft robot that can achieve self‐cycling forward crawling, Figure S33 (Supporting Information).

## 4 Experimental Section

### Materials of SRC and MLMD

A PDMS‐silica nanocomposite was prepared by mixing commercial silicone elastomer (Sylgard 184, Dow Corning Corporation) of 10:1 g (silicone base/curing agent) with 0.7 g of silica nanoparticles (Size: 7–40 nm, Sigma‐Aldrich, China) (Figure S4, Supporting Information) to form the programming and circular modules. The magnetic liquid metal (MLM) was prepared by mixing the liquid alloy (Galinstan, Geratherm Medical AG, Geschwenda, Germany) with commercial nano iron particles (Alibaba) and hydrochloric acid (Figure S7, Supporting Information) through manually rotating the 10 × 20 mm strong cylinder round magnets (Alibaba). The electrode modules were made of graphene film with 50 µm thickness (Best Materials Company, China).

### Preparation of SRC Modules

The electrode module was cut and surface modified by a UV‐laser marker (HGL‐LSU3/5EI, Huagong Laser, China, wavelength: 355 nm; power: 5 W; mode: pulse; beam quality: M2 = 1.2; laser intensity: 12.69 × 10^6^ W m^−2^) with different operating parameters (scan path, spacing, and speed), Figures S2 and S6 (Supporting Information). The programming and circular modules were made of PDMS‐silica nanocomposite by molding process with defined channel geometrics, and the surfaces were modified by the same UV‐laser marker above, Figure S4 (Supporting Information). The connection between each soft module was made of thermoplastic polyurethane printed by a 3D printer (UNION 3D Lite600), Figure S3 (Supporting Information).

### Characterization

The contact angles of the liquid metal droplet on different samples were measured with drop shape analysis equipment (DSA25, KRUSS, Hamburg, Germany), Figure 2C. The detailed micro surface morphology of electrodes and PDMS‐silica nanocomposite was measured by scanning electron microscopy on a GeminiSEM300 (Carl Zeiss, Germany), Figure 2B. The samples were sputter coated with gold before imaging, and the cross‐section image was directly cut from the sample surface, Figure S5 (Supporting Information). Energy dispersive X‐ray mappings of the MLMD were acquired by using an Oxford X‐Max EDS detector, Figure S11 (Supporting Information). The sliding motion of an MLMD was recorded by a high‐speed camera (Phantom v1212) and analyzed by phantom high‐speed software (PCC 3.6), Figure S14 (Supporting Information). The electrical performance of SRCs (resistance and output current) was tested by a precision LCR meter (E4980 AL) and a digital oscilloscope (Tektronix TBS 1202B‐EDU), respectively.

### Fabrication and Characterization of Soft Robotic Systems

The software Solidworks was used for designing 3D molds and for converting the designs into stereolithography (STL) files for 3D printing. The 3D printing was performed on a 3D printer (UNION 3D Lite600), using acrylonitrile‐butadiene‐styrene (ABS). All chemicals were purchased from the vendors and used as received, without further purification. Polydimethylsiloxane (PDMS) Sylgard 184 from Dow Corning. Ecoflex 30 and Dragon Skin 30 from Smooth‐On, Inc. Ultra‐high pull, nickel‐plated neodymium iron boron (NdFeB) ring magnets–were purchased from Alibaba. The current of SRC is recorded by Digital Oscilloscope (Tektronix TBS 1202B‐EDU) from two ends of electrodes with conducting MLMD. The output pressure is recorded by a pressure manometer (DP370) from Alibaba. The intersection of the car's contour diagonal as the feature point was selected, and its position change after each actuation of the bending actuators was manually measured. The position data are then plotted in Figure 5D, and the starting position of the feature point was fixed at the coordinate origin. More details about these soft robotic systems are introduced in Supporting Information.

## Conflict of Interest

The authors declare no conflict of interest.

## Supporting information

Supporting Information

Supplemental Movie 1

Supplemental Movie 2

Supplemental Movie 3

Supplemental Movie 4

Supplemental Movie 5

Supplemental Movie 6

Supplemental Movie 7

Supplemental Movie 8

Supplemental Movie 9

Supplemental Movie 10

## Data Availability

The data that support the findings of this study are available in the supplementary material of this article.

## References

[1] D. Rus , M. T. Tolley , Nature 2015, 521, 467. 26017446 10.1038/nature14543

[2] R. F. Shepherd , F. Ilievski , W. Choi , S. A. Morin , A. A. Stokes , A. D. Mazzeo , X. Chen , M. Wang , G. M. Whitesides , Proc. Natl. Acad. Sci. U. S. A. 2011, 108, 20400. 22123978 10.1073/pnas.1116564108 PMC3251082

[3] J. Zhu , Z. Chai , H. Yong , Y. Xu , C. Guo , H. Ding , Z. Wu , Soft Robot. 2023, 10, 30. 35584255 10.1089/soro.2021.0126

[4] A. D. Marchese , C. D. Onal , D. Rus , Soft Robot. 2014, 1, 75. 27625912 10.1089/soro.2013.0009 PMC4997624

[5] M. Wehner , R. L. Truby , D. J. Fitzgerald , B. Mosadegh , G. M. Whitesides , J. A. Lewis , R. J. Wood , Nature 2016, 536, 451. 27558065 10.1038/nature19100

[6] T. Umedachi , V. Vikas , B. Trimmer , Bioinspir. Biomim. 2016, 11, 025001. 26963596 10.1088/1748-3190/11/2/025001

[7] J. Zhu , M. Pu , H. Chen , Y. Xu , H. Ding , Z. Wu , Sci. China Technol. Sc. 2022, 65, 2156.

[8] Q. Xiao , X. Kuang , S. Wu , J. Wong , S. M. Montgomery , R. Zhang , J. M. Kovitz , F. Yang , H. J. Qi , R. Zhao , Adv. Mater. 2019, 32, 1906657. 10.1002/adma.201906657 31814185

[9] W. Hu , G. Z. Lum , M. Mastrangeli , M. Sitti , Nature 2018, 554, 81. 29364873 10.1038/nature25443

[10] X. Ke , S. Zhang , Z. Chai , J. Jiang , Y. Xu , B. Tao , H. Ding , Z. Wu , Mater. Today Phys. 2021, 17, 100313.

[11] T. T. Hoang , P. T. Phan , M. T. Thai , N. H. Lovell , T. N. Do , Adv. Mater. Technol. 2020, 5, 2000724.

[12] Y. F. Zhang , N. Zhang , H. Hingorani , N. Ding , D. Wang , C. Yuan , B. Zhang , G. Gu , Q. Ge , Adv. Funct. Mater. 2019, 29, 1806698.

[13] Z. Li , N. V. Myung , Y. Yin , Sci. Robot. 2021, 6, eabi4523. 34851711 10.1126/scirobotics.abi4523

[14] N. W. Bartlett , M. T. Tolley , J. T. B. Overvelde , J. C. Weaver , B. Mosadegh , K. Bertoldi , G. M. Whitesides , R. J. Wood , Science 2015, 349, 161. 26160940 10.1126/science.aab0129

[15] R. V. Martinez , J. L. Branch , C. R. Fish , L. Jin , R. F. Shepherd , R. M. Nunes , Z. G. Suo , G. M. Whitesides , Adv. Mater. 2013, 25, 205. 22961655 10.1002/adma.201203002

[16] J. Zhu , L. Lyu , Y. Xu , H. Liang , X. Zhang , H. Ding , Z. Wu , Adv. Intell. Syst. 2021, 3, 2100011.

[17] E. G. Hevia , L. De La Rochefoucauld , R. J. Wood , presented at 2022 IEEE Int. Conf. Robot. Autom. (ICRA) IEEE, PA, USA 2022, pp. 7138.

[18] D. Drotman , S. Jadhav , D. Sharp , C. Chan , M. T. Tolley , Sci. Robot. 2021, 6, eaay2627. 34043527 10.1126/scirobotics.aay2627

[19] B. H. Kim , K. Li , J.‐T. Kim , Y. Park , H. Jang , X. Wang , Z. Xie , S. M. Won , H.‐J. Yoon , G. Lee , W. J. Jang , K. H. Lee , T. S. Chung , Y. H. Jung , S. Y. Heo , Y. Lee , J. Kim , T. Cai , Y. Kim , P. Prasopsukh , Y. Yu , X. Yu , R. Avila , H. Luan , H. Song , F. Zhu , Y. Zhao , L. Chen , S. H. Han , J. Kim , et al., Nature 2021, 597, 503. 34552257 10.1038/s41586-021-03847-y

[20] W. Tang , Y. Lin , C. Zhang , Y. Liang , J. Wang , W. Wang , C. Ji , M. Zhou , H. Yang , J. Zou , Sci. Adv. 2023, 7, eabf8080. 10.1126/sciadv.abf8080 PMC8378814 34417171

[21] J. Liang , Y. Wu , J. K. Yim , H. Chen , Z. Miao , H. Liu , Y. Liu , Y. Liu , D. Wang , W. Qiu , Z. Shao , M. Zhang , X. Wang , J. Zhong , L. Lin , Sci. Robot. 2021, 6, eabe7906. 34193563 10.1126/scirobotics.abe7906

[22] W. Tang , C. Zhang , Y. Zhong , P. Zhu , Y. Hu , Z. Jiao , X. Wei , G. Lu , J. Wang , Y. Liang , Y. Lin , W. Wang , H. Yang , J. Zou , Nat. Commun. 2021, 12, 2247. 33854071 10.1038/s41467-021-22391-x PMC8046788

[23] G. Mao , D. Schiller , D. Danninger , B. Hailegnaw , F. Hartmann , T. Stockinger , M. Drack , N. Arnold , M. Kaltenbrunner , Nat. Commun. 2022, 13, 4456. 35945209 10.1038/s41467-022-32123-4 PMC9363453

[24] M. Wehner , R. L. Truby , D. J. Fitzgerald , B. Mosadegh , G. M. Whitesides , J. A. Lewis , R. J. Wood , Nature 2016, 536, 451. 27558065 10.1038/nature19100

[25] W.‐K. Lee , D. J. Preston , M. P. Nemitz , A. Nagarkar , A. K. MacKeith , B. Gorissen , N. Vasios , V. Sanchez , K. Bertodi , L. Mahadevan , G. M. Whitesides , Sci. Robot. 2022, 7, 63. 10.1126/scirobotics.abg5812 35138883

[26] M. Garrad , G. Soter , A. T. Conn , H. Hauser , J. Rossiter , Sci. Robot. 2019, 4, eaaw6060. 33137781 10.1126/scirobotics.aaw6060

[27] M. F. Simons , K. M. Digumarti , N. H. Le , H. Y. Chen , S. C. Carreira , N. S. S. Zaghloul , R. S. Diteesawat , M. Garrad , A. T. Conn , C. Kent , J. Rossiter , IEEE Robot. Autom. Lett. 2021, 6, 2.

[28] D. Li , T. Liu , J. Ye , L. Sheng , J. Liu , Adv. Intell. Syst. 2021, 3, 2000246.

[29] F. Li , J. Shu , L. Zhang , N. Yang , J. Xie , X. Li , L. Cheng , S. Kuang , S. Tang , S. Zhang , W. Li , L. Sun , D. Sun , Appl. Mater. Today 2020, 19, 100597.

[30] J. Shu , D. Ge , E. Wang , H. Ren , T. Cole , S. Tang , X. Li , X. Zhou , R. Li , H. Jin , W. Li , M. D. Dickey , S. Zhang , Adv. Mater. 2021, 33, 2103062. 10.1002/adma.202103062 34510575

[31] F. Li , S. Kuang , X. Li , J. Shu , W. Li , S. Tang , S. Zhang , Adv. Mater. Technol. 2019, 4, 1800694.

[32] X. Li , S. Li , Y. Lu , M. Liu , F. Li , H. Yang , S. Y. Tang , S. Zhang , W. Li , L. Sun , ACS Appl. Mater. Interfaces 2020, 12, 37670. 32700519 10.1021/acsami.0c08179

[33] E. J. Markvicka , M. D. Bartlett , X. Huang , C. Majidi , Nat. Mater. 2018, 17, 618. 29784995 10.1038/s41563-018-0084-7

[34] R. Guo , X. Sun , B. Yuan , H. Wang , J. Liu , Adv. Sci. 2019, 6, 1901478. 10.1002/advs.201901478 PMC6794621 31637174

[35] A. Zavabeti , J. Z. Ou , B. J. Carey , N. Syed , R. Orrell‐Trigg , E. L. H. Mayes , C. Xu , O. Kavehei , A. P. O'Mullane , R. B. Kaner , K. Kalantar‐zadeh , T. Daeneke , Science 2017, 358, 332. 29051372 10.1126/science.aao4249

[36] A. Elbourne , S. Cheeseman , P. Atkin , N. P. Truong , N. Syed , A. Zavabeti , M. Mohiuddin , D. Esrafilzadeh , D. Cozzolino , C. F. McConville , M. D. Dickey , R. J. Crawford , K. Kalantar‐Zadeh , J. Chapman , T. Daeneke , V. K. Truong , ACS Nano 2020, 14, 802. 31922722 10.1021/acsnano.9b07861

[37] L. Hu , L. Wang , Y. Ding , S. Zhan , J. Liu , Adv. Mater. 2016, 28, 9210. 27571211 10.1002/adma.201601639

[38] Y. Ding , M. Zeng , L. Fu , Matter 2020, 3, 1477.

[39] I. Joshipura , H. Ayers , G. Castillo , C. Ladd , C. Tabor , J. Adams , M. Dickey , ACS Appl. Mater. Interfaces 2018, 10, 44686. 30532957 10.1021/acsami.8b13099

[40] Y. Xu , P. Deng , G. Yu , X. Ke , Y. Lin , X. Shu , Y. Xie , S. Zhang , R. Nie , Z. Wu , Sens. Actuators B Chem. 2021, 343, 130085.

[41] S. Zhang , Q. Jiang , Y. Xu , C. F. Guo , Z. Wu , Micromachines 2020, 11, 682. 32674399 10.3390/mi11070682 PMC7407878

[42] Y. Xu , B. Jiang , Int. J. Adv. Manuf. Tech. 2021, 113, 883.

[43] J. De Vicente , D. J. Klingenberg , R. Hidalgo‐Alvarez , Soft Matter 2011, 7, 3701.

[44] F. Carle , K. Bai , J. Casara , K. Vanderlick , E. Brown , Phys. Rev. Fluids. 2017, 2, 013301.

[45] K. Doudrick , S. Liu , E. M. Mutunga , K. L. Klein , V. Damle , K. K. Varanasi , K. Rykaczewski , Langmuir 2014, 30, 6867. 24846542 10.1021/la5012023

[46] T. Koishi , K. Yasuoka , S. Fujikawa , T. Ebisuzaki , X. C. Zeng , Proc. Natl. Acad. Sci. U. S. A. 2009, 106, 8435. 19429707 10.1073/pnas.0902027106 PMC2688995

[47] V. Sivan , S. Tang , A. P. Mullane , P. Petersen , N. Eshtiaghi , K. K. Zadeh , A. Mitchell , Adv. Funct. Mater. 2012, 23, 144.

[48] L. Ren , S. Sun , G. Casillas , M. Nancarrow , G. Peleckis , M. Turdy , K. Du , X. Xu , W. Li , L. Jiang , S. Dou , Y. Du , Adv. Mater. 2018, 30, 1802595. 10.1002/adma.201802595 30015992

[49] M. D. Dickey , R. C. Chiechi , R. J. Larsen , E. A. Weiss , D. A. Weitz , G. M. Whitesides , Adv. Funct. Mater. 2008, 18, 1097.

[50] T. Daeneke , K. Khoshmanesh , N. Mahmood , I. A. de Castro , D. Esrafilzadeh , S. J. Barrow , M. D. Dickey , K. Kalantar‐zadeh , Chem. Soc. Rev. 2018, 47, 4073. 29611563 10.1039/c7cs00043j

[51] X. Fan , X. Dong , A. C. Karacakol , H. Xie , M. Sitti , Proc. Natl. Acad. Sci. U. S. A. 2020, 117, 27916. 33106419 10.1073/pnas.2016388117 PMC7668164

[52] S. Fusco , M. S. Sakar , S. Kennedy , C. Peters , R. Bottani , F. Starsich , A. Mao , G. A. Sotiriou , S. Pané , S. E. Pratsinis , D. Mooney , B. J. Nelson , Adv. Mater. 2014, 26, 952. 24510666 10.1002/adma.201304098

[53] S. Xu , Y. Chen , N. P. Hyun , K. P. Becker , R. J. Wood , Proc. Natl. Acad. Sci. U. S. A. 2021, 118, e2103198118. 34417289 10.1073/pnas.2103198118 PMC8403939

[54] N. D. Kohls , R. Balak , B. P. Ruddy , Y. C. Mazumdar , Soft Robot. 2023. 10.1089/soro.2022.0075 36976757

[55] N. Kohls , I. Abdeally , B. P. Ruddy , Y. C. Mazumdar , ASME Letters Dyn. Syst. Contr. 2021, 1, 031011.

[56] T. N. Do , H. Phan , T. Q. Nguyen , Y. Visell , Adv. Funct. Mater. 2018, 28, 1800244.

[57] R. Balak , Y. C. Mazumdar , IEEE Robot. Autom. Lett. 2021, 6, 8285.

