# Correction to “A Soft Reconfigurable Circulator Enabled by Magnetic Liquid Metal Droplet for Multifunctional Control of Soft Robots”

**DOI:** 10.1002/advs.202401071

**Published:** 2024-03-09

**Authors:** Yi Xu, Jiaqi Zhu, Han Chen, Haochen Yong, Zhigang Wu

A Soft Reconfigurable Circulator Enabled by Magnetic Liquid Metal Droplet for Multifunctional Control of Soft Robots. *Adv. Sci*. **2023**, *10*, 2300935

In the part of the “Acknowledgements” section, the text “The work was funded by the National Science Foundation of China (nos. 52188162 and U1613204)” was incorrect. This should read: “The work was funded by the National Science Foundation of China (nos. 52188102 and U1613204)”

We apologize for this error.

