# Supplementary material for: A Soft Reconfigurable Circulator Enabled by Magnetic Liquid Metal Droplet for Multifunctional Control of Soft Robots
Source: Adv Sci (Weinh). 2023 Jun 13;10(23):2300935. doi: 10.1002/advs.202300935 (PMC10427373; doi:10.1002/advs.202300935)
Supplement: Supplementary file 1 — Supporting Information [file ADVS-10-2300935-s001.pdf]

ADVANCED SCIENCE
Open Access

Supporting Information

for *Adv. Sci.*, DOI 10.1002/advs.202300935

A Soft Reconfigurable Circulator Enabled by Magnetic Liquid Metal Droplet for Multifunctional Control of Soft Robots

*Yi Xu, Jiaqi Zhu, Han Chen, Haochen Yong and Zhigang Wu**

Support Information

A soft reconfigurable circulator enabled by magnetic liquid metal droplet for multifunctional control of soft robots

*Yi Xu †, Jiaqi Zhu †, Han Chen †, Haochen Yong, Zhigang Wu**

Soft Intelligence Lab, State Key Laboratory of Digital Manufacturing Equipment and Technology, Huazhong University of Science and Technology, Wuhan 430074, China

† These authors contributed equally to this work.

*Correspondence: Email: zgwu@hust.edu.cn (Z.W.)

Fabrication and characterization of various soft robotic systems

Electrically conductive soft grippers: The modular soft gripper consisted of three detachable soft fingers and one soft base (Figure S23). Both the fingers and the base (Dragon skin 20) were made by multi-step molding, and the soft fingers were assembled by gluing a stretchable layer (Ecoflex 30) and a strain-limiting layer (Dragon skin 20) together. The fingers and the base can be assembled together into a soft pneumatic gripper to conduct various grasping tasks, and the connecting method involves several ring magnets embedded under the soft interfaces is similar to the previous study.[1] Moreover, two complementary types of strip copper sheets were separately attached to the connecting surfaces of the fingers and the base (Figure S23). Only when the finger is aligned and successfully assembled onto the base, the circuit can be functioned well.

Soft car: The soft vehicle mainly consists of two pneumatic bending actuators, one soft main body with embedded soft circulator, one integrated pump (EDZP02-D3, 0.06MPA), two integrated solenoid valves (DGLJ5503), and four one-way wheels (Figure 5A, left panel). All the soft components of the car were fabricated by molding. The two bending actuators were made by gluing a stretchable layer (Ecoflex 30) and a strain-limiting layer (Dragon skin 20) together. They shared the same integrated air pump and were separately controlled by two integrated solenoid valves controlled by the soft circulator embedded in the soft main body (PDMS). The bending actuators were distributed on both sides of the soft main body. Each of the four one-way wheels was composed of a rubber ring (made of Ecoflex 30), a one-way bearing (OWC 814GXLZ) and an axle (3D printed using WEILAI 8100 resin), and was

assembled into the main body through several supporting bearings (YXVSY-604). Through the periodic actuation of the two bending actuators, the soft car can twist or arch its body to move forward in various trajectories. During the moving process, the four wheels can be selectively stuck, transferring the deformation of the soft car into its body movement (Figure S24).

Soft sorting gripper: The soft sorting gripper mainly consisted of a soft arm, a soft gripper, a flexible sensor, and several soft connectors (Supplementary Fig. S26). All these components were fabricated by molding. Among them, both the soft arm and the soft gripper were made by gluing a stretchable layer (Ecoflex 30) and a strain-limiting layer (Dragon skin 20) together. The flexible sensor was fabricated by fixing the graphene film electrodes and the copper lines onto the inner surface of the soft beam structure (Ecoflex 30, Supplementary Fig. S27). The soft arm was fixed on the rigid frame through a soft connector (Dragon skin 20) with two reserved through holes. The soft arm and the soft gripper were glued together through two soft connectors (Dragon skin 20), and the air tube was inserted into the cylindrical soft connector to provide air supply for the soft gripper. The flexible sensor was attached to the center of the soft gripper for the detection of the press forces. During the sorting process, the soft arm gradually bent and successively lifted the soft gripper to a higher position for object grasping. When the soft gripper successfully grasped an object, the flexible sensor would be pressed to trigger the relaxation motion of the arm and gripper in order.

Supporting Table:

Reference	Computing medium	Input simplicity	Functional diversity	Structural reusability	Compatibility
Jin *et al.* [2]	Air pressure	Low (intermittent air pressure with different values)	Low (programming only)	Low (partial redesign is needed to change the program)	Compatible only with pneumatic systems
van Laake *et al.* [3]		High (constant pressure signal)	Low (programming only)	High (reprogramming can be achieved by rewiring)	
Lee *et al.* [4]		High (constant pressure signal)	Intermediate (feedback control & programming)	High (reprogramming can be achieved by rewiring)	
Rothemund *et al.* [5]		High (constant pressure signal)	Intermediate (simple feedback control & programming)	Intermediate (partial redesign is needed to achieve different control functions)	
Decker *et al.* [6]		High (constant pressure signal)	Relatively high (partial types of logic gates & programming & feedback control)	Intermediate (partial redesign is needed to achieve different control functions)	
Garrad *et al.* [7]	Conducting liquid segment	High (constant pressure signal)	Relatively high (partial types of logic gates & programming & feedback control)	Intermediate (partial redesign is needed to achieve different control functions)	Compatible with various electronic-based systems
Li *et al.* [8]	Liquid metal droplet	Intermediate (different voltage values)	Low (partial types of logic gates)	High (two modules can construct various logic gates)	
This work		High (reciprocating magnetic signal)	High (all types of logic gates & programming & self-adaptive control (a combination of programming and feedback control))	High (different control functions can be realized by re-configuring the same modules)	

Table S1. Comparison of several soft fluidic control circuits

Supporting Figures:

Figure S1. Soft machines with rigid electronic components.
Figure S2. Electrodes attaching process onto programming modules.
Figure S3. Connecting methods between each soft module.
Figure S4. Schematic illustration of soft material surface modification.
Figure S5. Microstructure and silica exposure control of soft composite.
Figure S6. Ultraviolet laser micro texturing on the graphene film surface.
Figure S7. Schematic illustration of the magnetic liquid metal (MLM) preparation.
Figure S8. Various observed phenomena when the suspension of iron particles in Galinstan.
Figure S9. Confined the wall force and magnetic force enable circular movement of MLMD.
Figure S10. Schematic illustration of different behaviors of MLMD with obstacles in the micro-channels.
Figure S11. Energy dispersive X-ray (EDX) mapping of split MLMD.
Figure S12. Schematic description of the DEA-based soft gate.
Figure S13. DEA-based soft gates for mode switching associated with programming module (yellow).
Figure S14. Sliding of an MLMD in the presence of a moving permanent magnet.
Figure S15. Geometrical parameters design of electrodes and MLMD for conducting behaviors.
Figure S16. Single computing unit operates in either digital or analog mode.
Figure S17. Shape deformability of MLMD under different magnetic flux densities.
Figure S18. Schematic description of graphene film electrodes processing.
Figure S19. Schematic of a single computing unit performing pulse-width modulated (PWM) outputs.
Figure S20. Final output of a full set of logic gates
Figure S21. Schematic of serial and parallel electrode connections achieving a full set of soft logic gates.
Figure S22. Conducting criterion of the soft finger.
Figure S23. Assembling electrically conductive soft fingers.
Figure S24. Schematic of working principle of different locomotion controlled by reconfiguration of fundamental modules.
Figure S25. Structural design and actuation mechanism of the soft arm and gripper.
Figure S26. A flexible sensor for information storage.
Figure S27. Program design with reconfigurable modules for self-adaptive control.

Figure S28. Schematic of a manual magnetic actuation method.

Figure S29. An SRC-based soft manual control switch.

Figure S30. Schematic of a magnetic actuation method based on soft electromagnets to achieve fully onboard SRC systems without external input magnetic signals.

Figure S31. Schematic of a self-cyclic SRC-based screening oscillator under gravity.

Figure S32. The screening oscillator can automatically achieve the screening of large and small spheres within 6 s by self-cyclic oscillation.

Figure S33. Schematic of the automatic crawling of a quadruped soft robot controlled by an SRC-based gravity-driven oscillator.

Supporting Movies:

Movie S1. Non-Wetting characteristics of magnetic liquid metal droplet outside liquid environment.

Movie S2. The conductivity and extreme deformation of MLMD for basic computing information.

Movie S3. A full set of logic gates enabled by MLMD.

Movie S4. Information storage for constructing logic circuits.

Movie S5. Computing process of a logic circuit for grasping function diagnosis.

Movie S6. Reconfiguration of basic modules for programming a soft car.

Movie S7. Self-adaptive control of a soft gripper as a sorting robot.

Movie S8. Cyclic motion of the MLMD driven by rigid electromagnets.

Movie S9. Cyclic motion of the MLMD driven by soft electromagnets.

Movie S10. Cyclic motion of the MLMD driven by gravity.

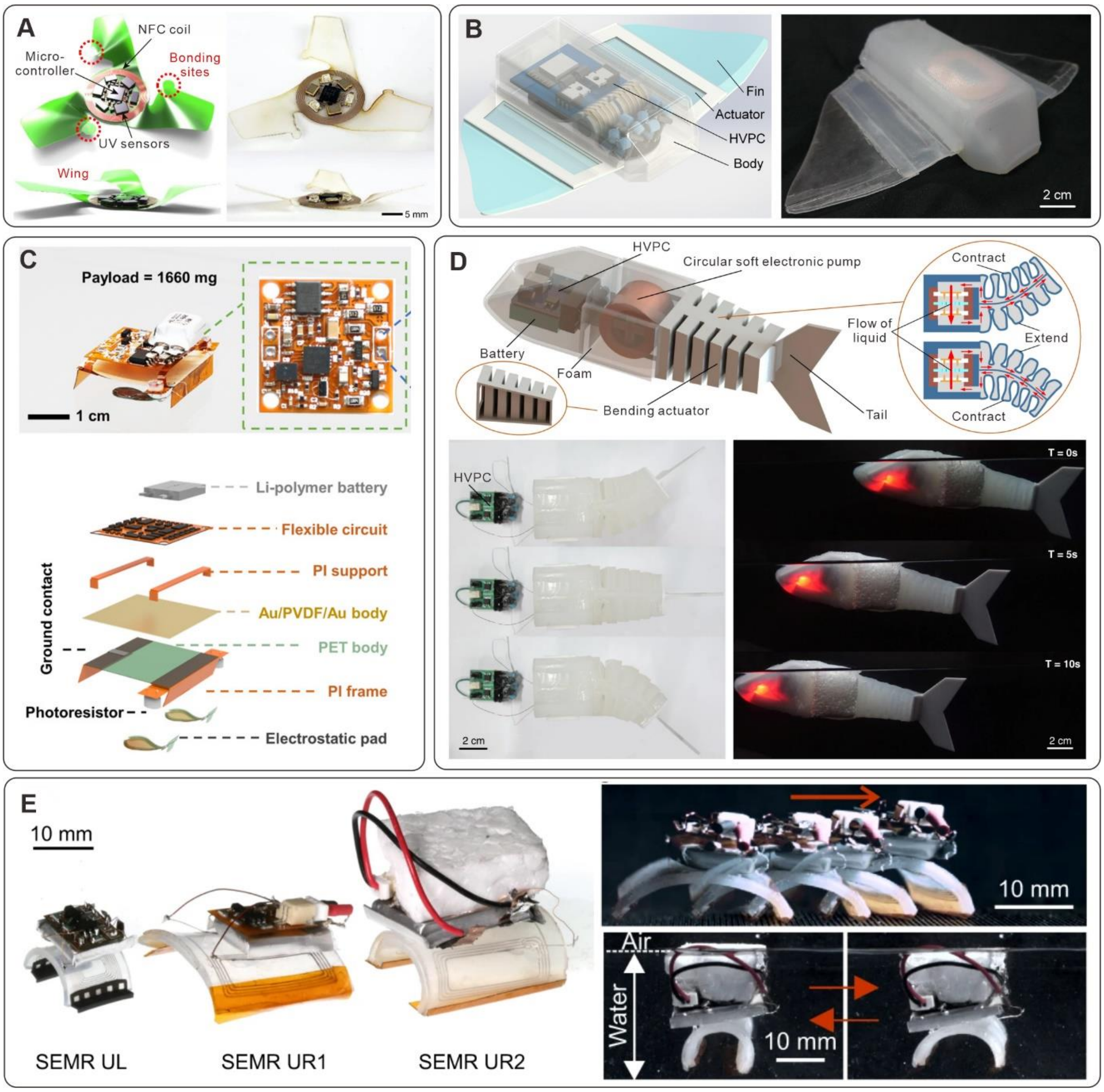

Figure S1. Soft machines embedded with rigid electronic components. (A) Three-dimensional electronic microfliers inspired by wind-dispersed seeds.[9] (B) Self-contained soft electrofluidic actuators.[10] (C) Electrostatic footpads enable agile insect-scale soft robots with trajectory control.[11] (D) Customizing a self-healing soft pump for a robot.[12] (E) Ultrafast small-scale soft electromagnetic robots.[13]

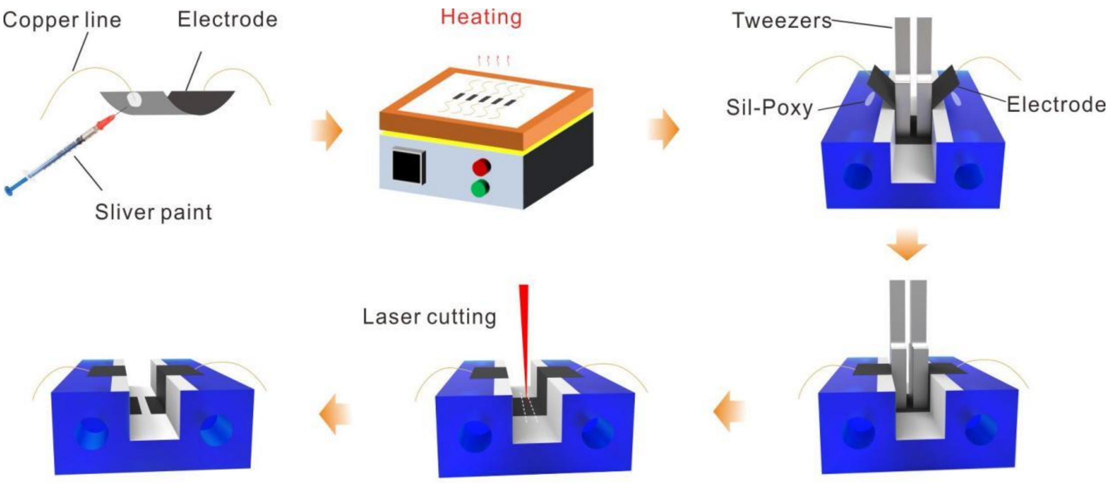

Figure S2. The graphene film electrode can be firmly attached onto the micro-channels with silpoxy and tweezers.

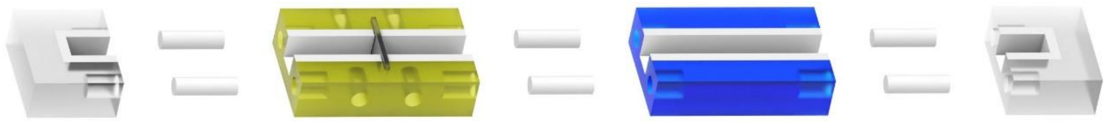

Figure S3. Soft modules are connected to each other by thermoplastic polyurethane (soft connectors). Therefore, the modules can be easily reconfigured to form new SRCs.

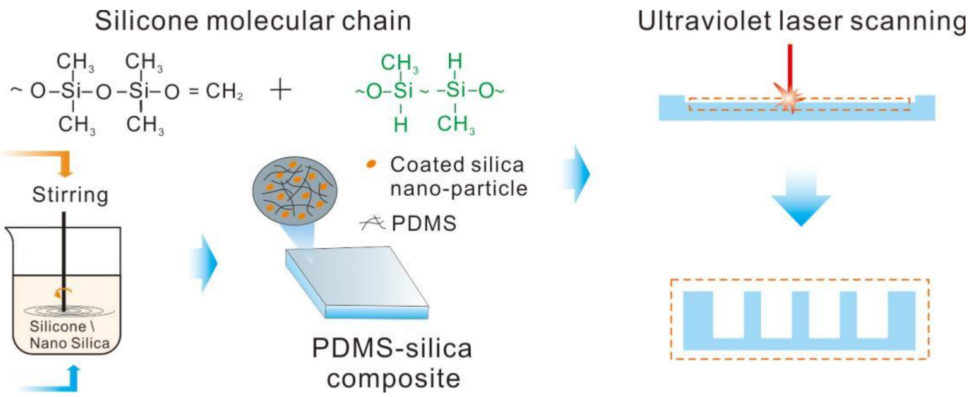

Figure S4. Schematic illustration of soft material surface modification. The PDMS-silica composite was prepared by stirring and curing process. After that, an optimized micromachining path of ultraviolet was chosen to scan on the soft material surface. Therefore, a column-like array surface can be achieved.

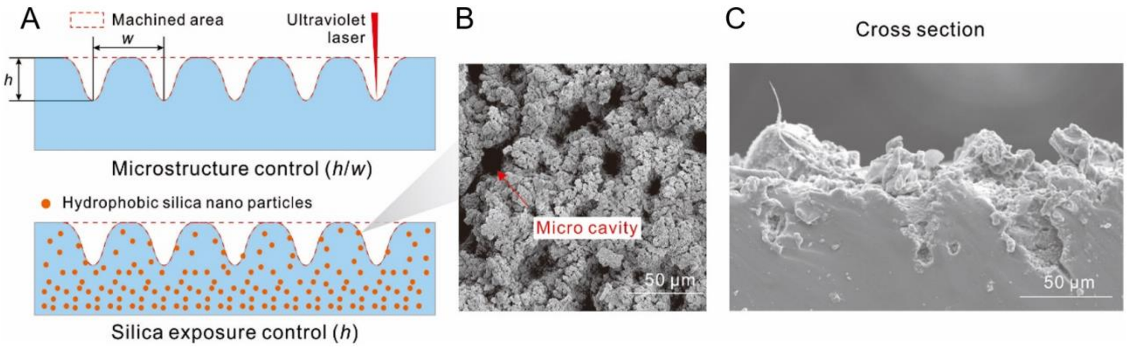

Figure S5. Microstructure and silica exposure control of soft composite (A) Schematic illustration of silica nanoparticles exposure control after UV laser micromachining. The aspect ratio (*h/w*) is depending on the machining parameters of the ultraviolet laser. The deeper the machining depth achieves, the more silica nanoparticles expose. (B) SEM image of modified surface. (C) Cross section of modified surface.

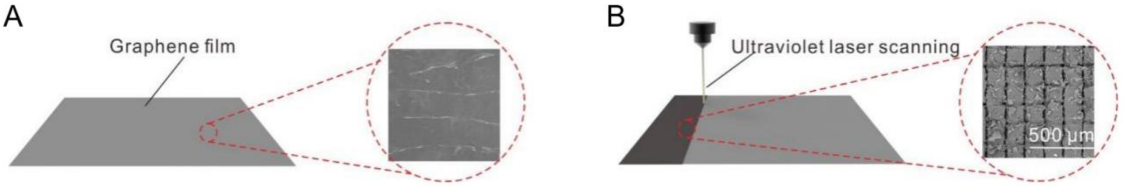

Figure S6. Ultraviolet laser micro texturing on the graphene film surface. (A) SEM image of untextured surface. (B) SEM image of the textured surface.

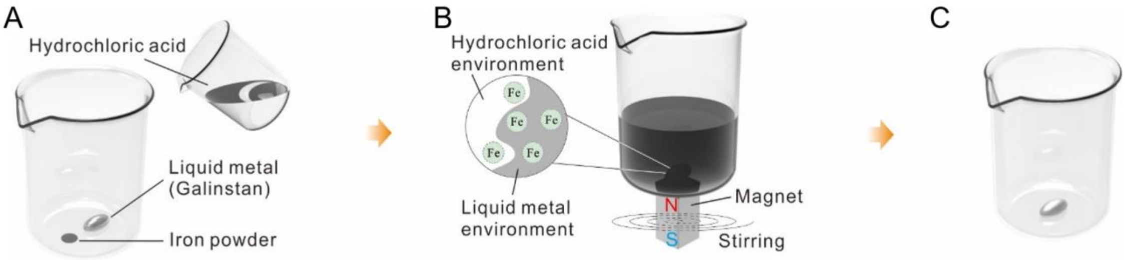

Figure S7. Schematic illustration of the magnetic liquid metal (MLM) preparation. (A) The MLM was made by mixing the Galinstan (typically 2 mL) with different volume fractions of iron particles and 12 mol/L HCl solution at room temperature. (B) Enough HCl was used to completely cover the metals preventing contact between the particles and air (typically 8 mL HCl for 2 mL of liquid metal). (C) Magnetic flux stirring makes the iron particles that came into contact with the liquid metal absorbed and suspended into the bulk within a few seconds.

By vigorous shaking and stirring of the solution for several min (after the HCl solution became transparent.), the MLM can be achieved by pouring out the solutions.

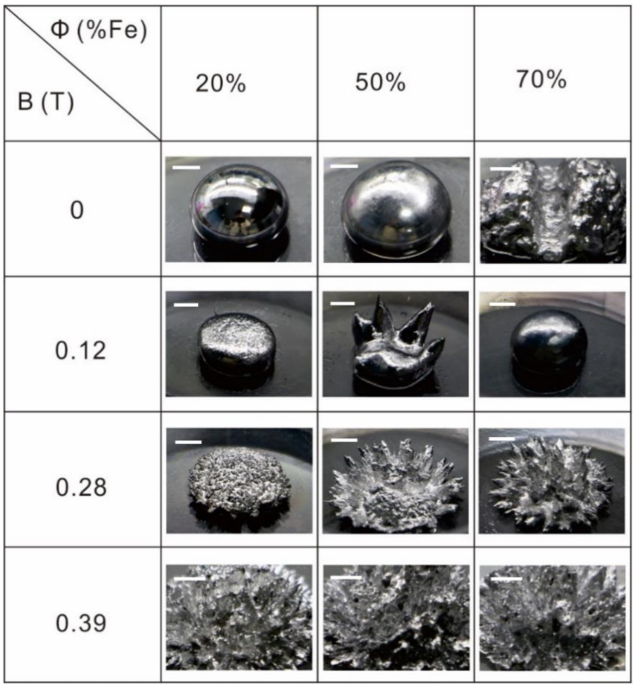

Figure S8. Various observed phenomena when the suspension of iron particles in Galinstan, at different value fractions Φ and applied constant inhomogeneous magnetic fields. A suspension at Φ= 20%, the MLM appears similar to the pure Galinstan. However, the MLM becomes rigid when increasing the iron particle fractions. The magnetorheological effect can be observed when the fraction is 20% at a magnetic field of 0.28 T. The Rosensweig instability can be obviously observed at about 0.39 T. Liquid metal states appear shiny since they are conductive and have a smooth surface due to the surface tension of the liquid, while solid states appear rough and less shiny since the particles poke through the liquid-air surface. Scale bars, 2 mm.

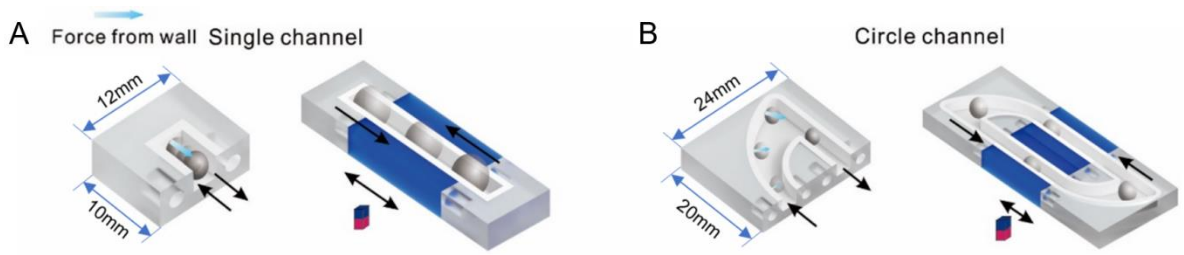

Figure S9. Confined the wall force and magnetic force enable circular movement of MLMD under reciprocating motion of magnetic flux. The width and length of the programming module are 12 mm and 20 mm, respectively. For larger circular modules, the width and length

are 24 mm and 20 mm, respectively, while for smaller circular modules they are 12 mm and 10 mm, respectively. (A) reciprocating circular movement. (B) one-way circular movement.

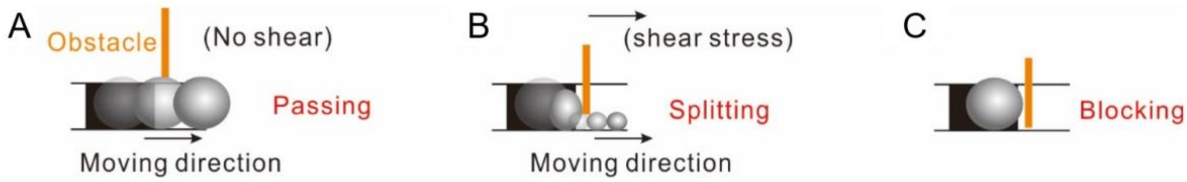

Figure S10. Schematic illustration of different behaviors of MLMD with obstacles in the micro-channels. (A) When the hole is large enough, the MLMD can easily pass through. (B) When the hole is very narrow, high shear stress will be caused by the obstacle to split the MLMD into two. (C) When the obstacle totally blocks the way, the MLMD cannot pass through and will be blocked.

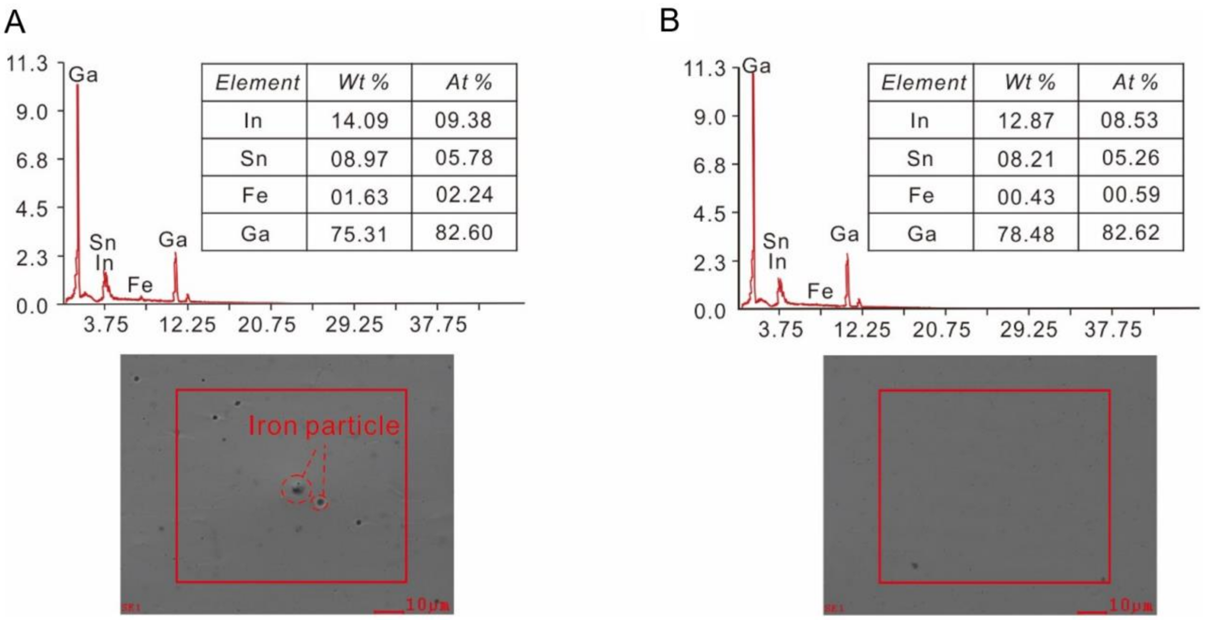

Figure S11. Energy dispersive X-ray (EDX) mapping of split MLMD. (A) Iron particles can be detected with 1.63% weight fraction ratio from one split MLMD. (B) Seldom iron particles can be found from another split MLMD.

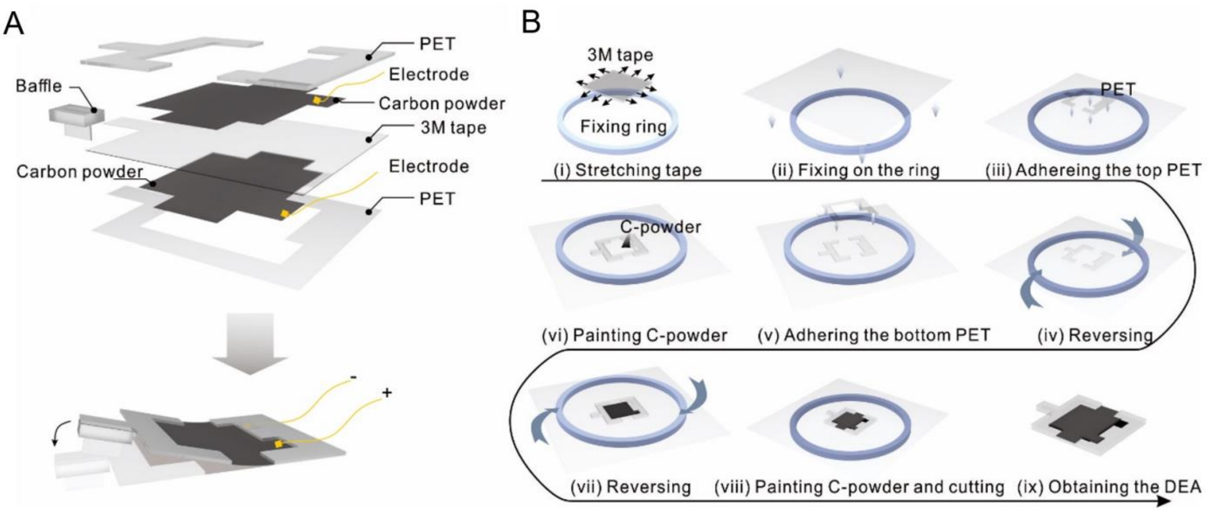

Figure S12. Schematic description of the DEA-based soft gate (A) The DEA-based soft gate is designed with the underlying principle of the lowest possible energy. The shape of the actuating structure is obtained as a re-organization of the overall structure until a configuration with the lowest possible energy is achieved. (B) The simple actuating structure with minimum energy is applied as a soft gate. When the actuator is released from the pre-stretching frame, it keeps on the shape with minimum energy. While a voltage is applied, the actuators are bending in a controlled manner, exhibiting a flat shape at around 3.5 kV.

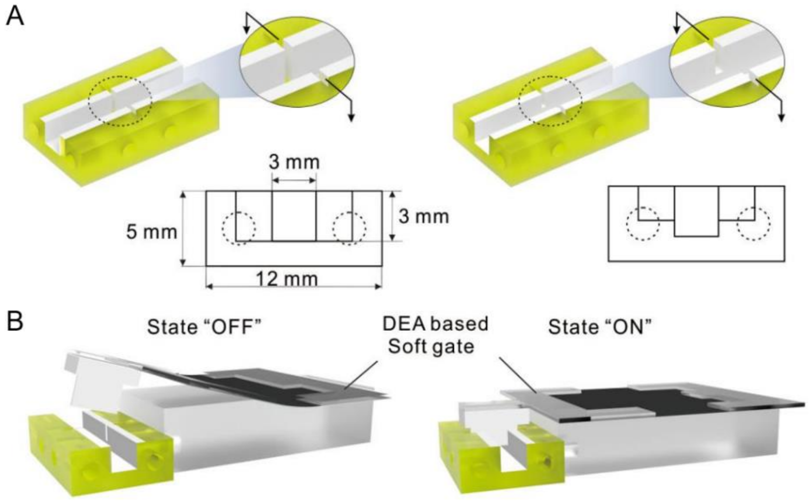

Figure S13. Two typical yellow programming modules and their associated DEA-based soft gates. (A) The cross sections show that the key difference between the two yellow programming modules is the depth of the groove perpendicular to the channel (magnification view). The groove of the left side module is the same height as the bottom of the channel, which can be used to control whether the MLMD is blocked or not. The groove of the right-side module is higher than the bottom of the channel, which can be used to control whether the MLMD is split or not. (B) The grooves are cooperated with the soft DEA actuator that can

be regarded as an on/off gate to decide whether the MLMD can be split, blocked or passed. Therefore, the DEA-based soft gate integrated with the soft programming module (yellow) can be regarded as a program switching module, e.g., when MLMD is conducting the electrodes, the actuators will be activated to execute program one. As the splitting behavior is triggered, MLMD with seldom iron will stay still to maintain the program one on while another splitting MLMD with iron particles will move to conduct other electrodes executing the program in sequence.

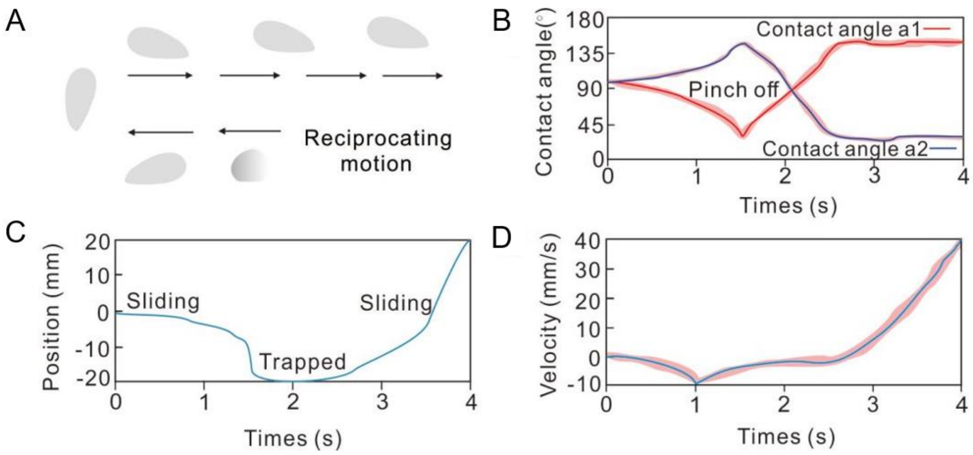

Figure S14. Sliding of an MLMD in the presence of a moving permanent magnet (B= 120 mT, v= 40 mm/s) (A) Evolution of the moving MLMD during reciprocating motion. The time-dependent image of the MLMD moves from the middle to the left and then right (Reciprocating motion). (B) Contact angles versus time. The approaching magnet pulls the droplet to the left, therefore, the droplet deforms so that the contact angle a1 will decrease from 93º to 35º. Then, the magnet goes to the right. However, the droplet is still in the stationary state at the pinch-off moment, since the magnetized iron particles will change direction according to the magnetic flux alternation. The solid/liquid/gas contact line is displaced and the contact angle a1 increases to 97°. The resultant force of the friction and the magnetic force equal to zero at such a moment. (C) Displacement of the MLMD versus time. A clear trapping zone can be observed during the reciprocating motion. In this process, the relative distance between the magnet and the droplet is adjusted to an equilibrium between friction force and magnetic force. (D) Velocity of the MLMD versus time. The droplet velocity will accelerate to catch the motion speed of the magnet to a constant speed in the sliding zone, e.g., 40 mm/s in our experiments, and keeps at a constant contact angle of 147°.

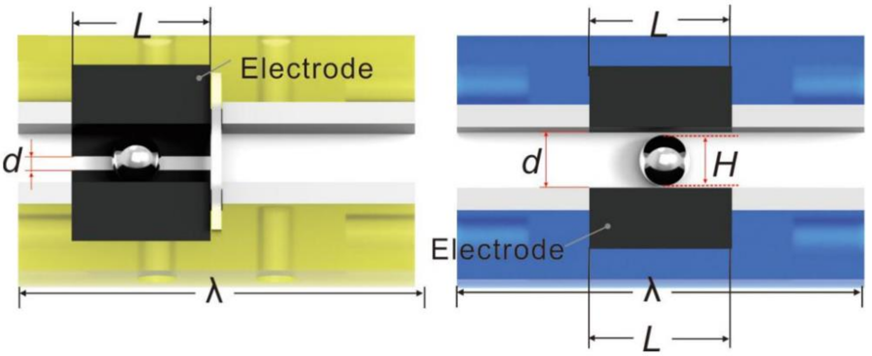

Figure S15. Geometrical parameters design of electrodes and MLMD for conducting behaviors. In computational terms, the geometrical parameter *H* of MLMD, the electrode length *L,* and the gap between electrodes *d*, are the key parameters which decide the conducting behavior between MLMD and electrodes.

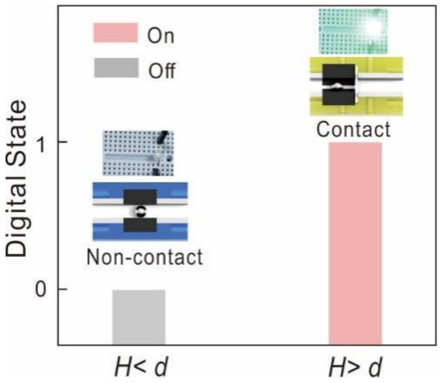

Figure S16. Single computing unit operates in either digital or analog mode with relative geometrical parameters (*H* and *d*). In digital mode, a programming module outputs "0" when the MLMD is not connecting with two sides of the graphene electrode (*H*<*d*) as state "Off". Else, the module outputs "1" as state "On"

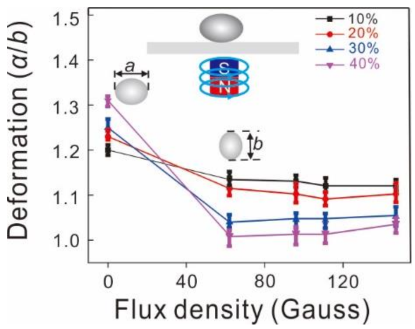

Figure S17. Shape deformability of MLMD under different magnetic flux density.

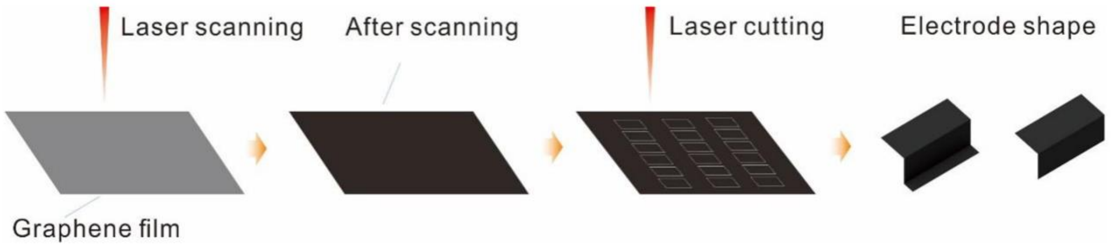

Figure S18. Schematic description of graphene film electrodes processing. Two typical electrodes are designed and can be easily fabricated by ultraviolet laser scanning and cutting process under different machining parameters.

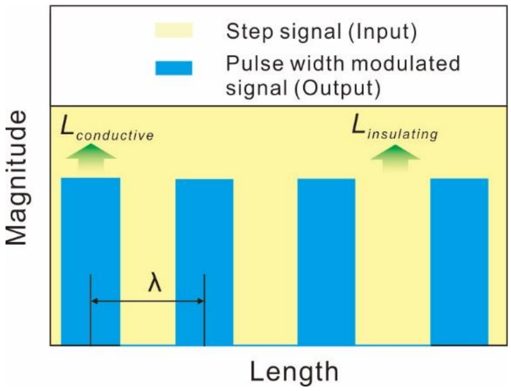

Figure S19. Schematic of a single programming module that can perform the analog computation to produce pulse-width modulated (PWM) outputs. The input signals (the circular motion of MLMD at constant speed) can be either amplified or filtered with predefined electrode length L and gap between electrodes d, allowing a wide range of input-output mappings for the programming design. The length of a programming module is defined as wavelength λ, and the input fraction D_{in} is decided by the length of a single electrode L, denoted as $D_{in}=L/\lambda$. As a consequence, a repeating signal with a conductive length of $L_{conductive}=\lambda D_{in}$ and an insulating length of $L_{insulating}=\lambda(1\text{-n}D_{in})$ can be treated as pulse-width modulated (PWM) outputs.

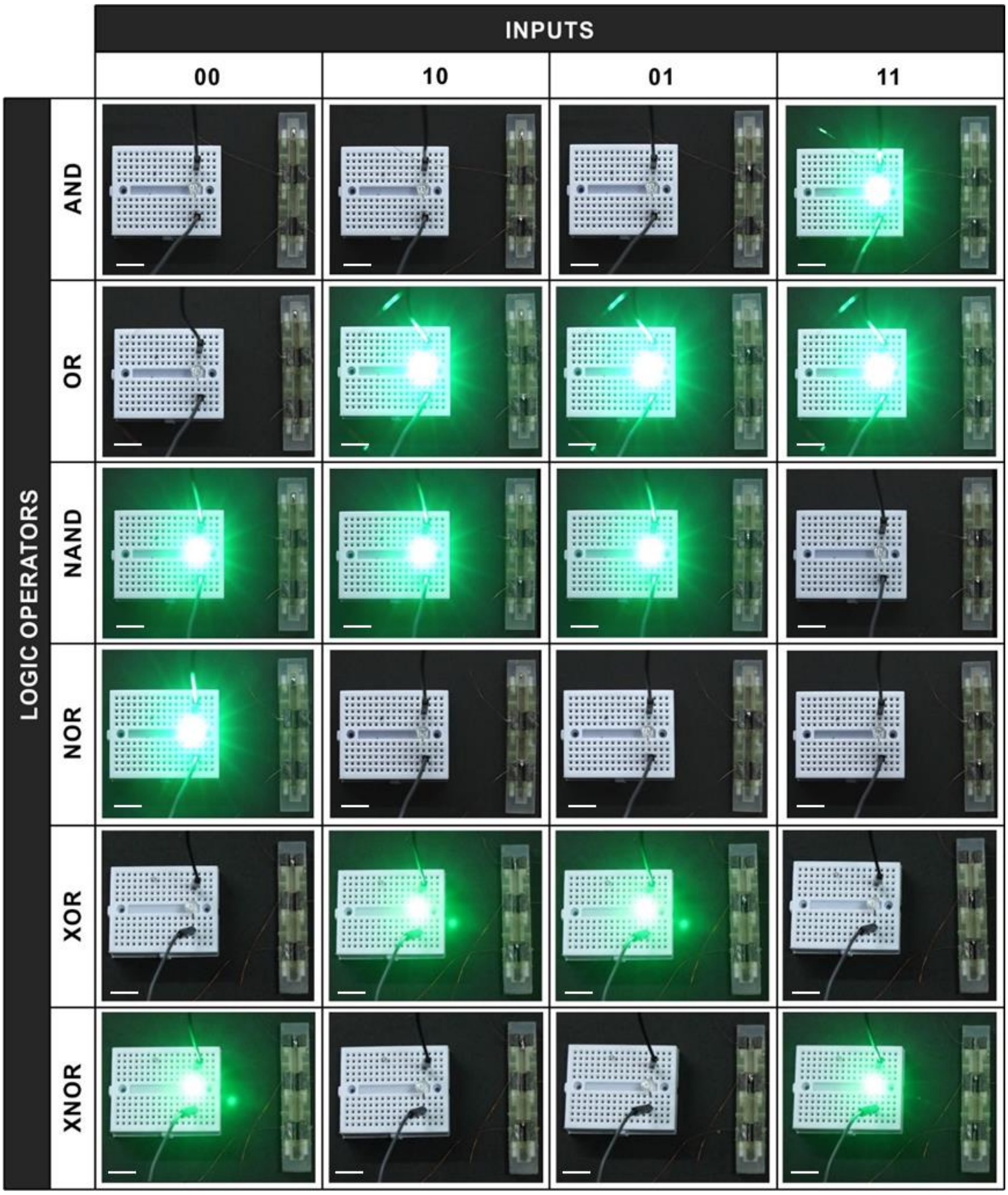

Figure S20. Final output of a full set of various logic gates: AND, OR, NAND, NOR, XNOR, XOR. Truth table "1" output is demonstrated with the LED lights up, else with LED lights off. Scale bars, 1 cm.

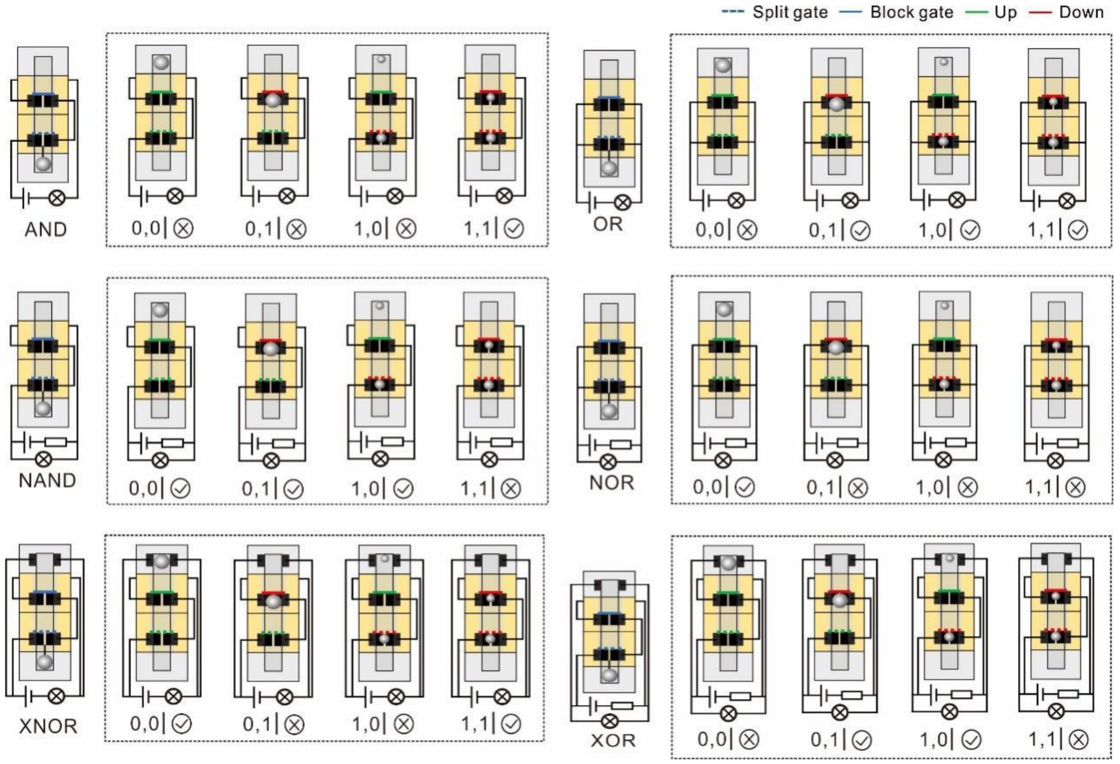

Figure S21. Schematic of serial and parallel connections with programming modules (yellow) that can achieve a full set of the soft logic gates: AND, OR, NAND, NOR, XNOR, XOR.

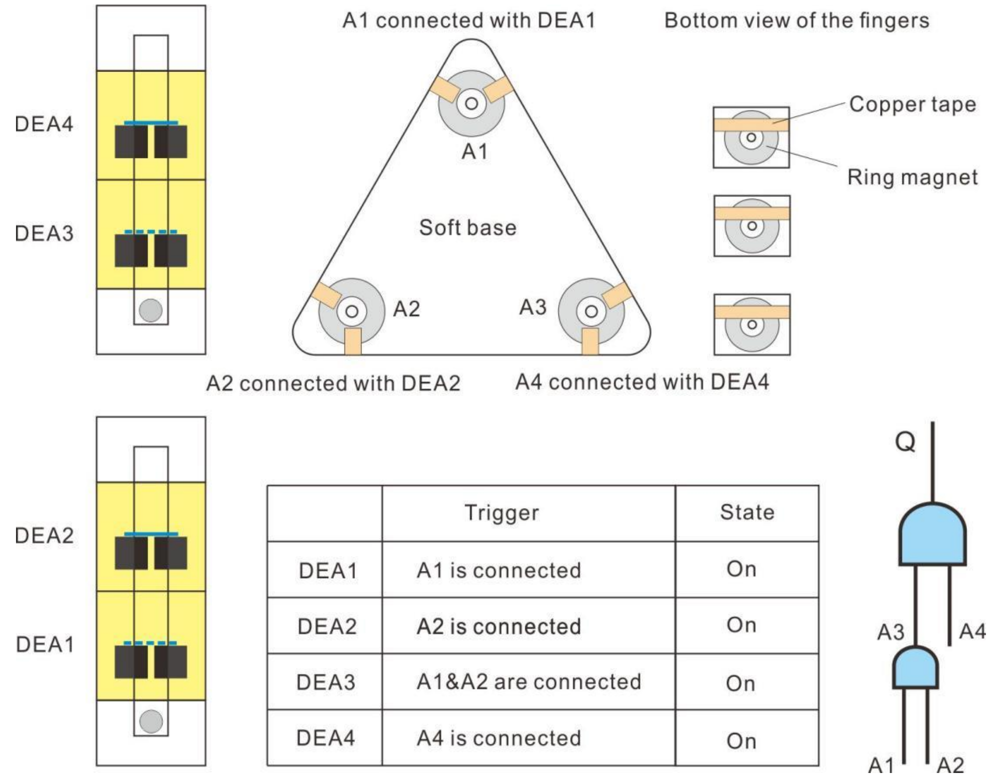

	Trigger	State
DEA1	A1 is connected	On
DEA2	A2 is connected	On
DEA3	A1&A2 are connected	On
DEA4	A4 is connected	On

Figure S22. The conducting criterion. Each of the magnets from the soft base and the bottom of the soft fingers is both attached with copper tape. Once the soft finger is correctly assembled onto the soft base, copper tapes are contacted to trigger the activation of DEA

based soft gate. Noting that the state of DEA3 is dependent on the output state of DEA1 and DEA2. The assembling information can be stored as DEA-based gate bending information. As a consequence, the gate "on" and "off" state can be stored in the soft programming module (yellow) depending on the assembling mode (correctly or falsely assembled) or orders (A1, A2 or A3). Once the assembling information is stored with the correct position, e.g. A1, A2, A3 are all assembled correctly. The state of DEA1 and DEA2 are output "1", and DEA3 and DEA4 also output "1" too. As a result, the gripper will be activated to grasp when the computing process is finished with MLMD.

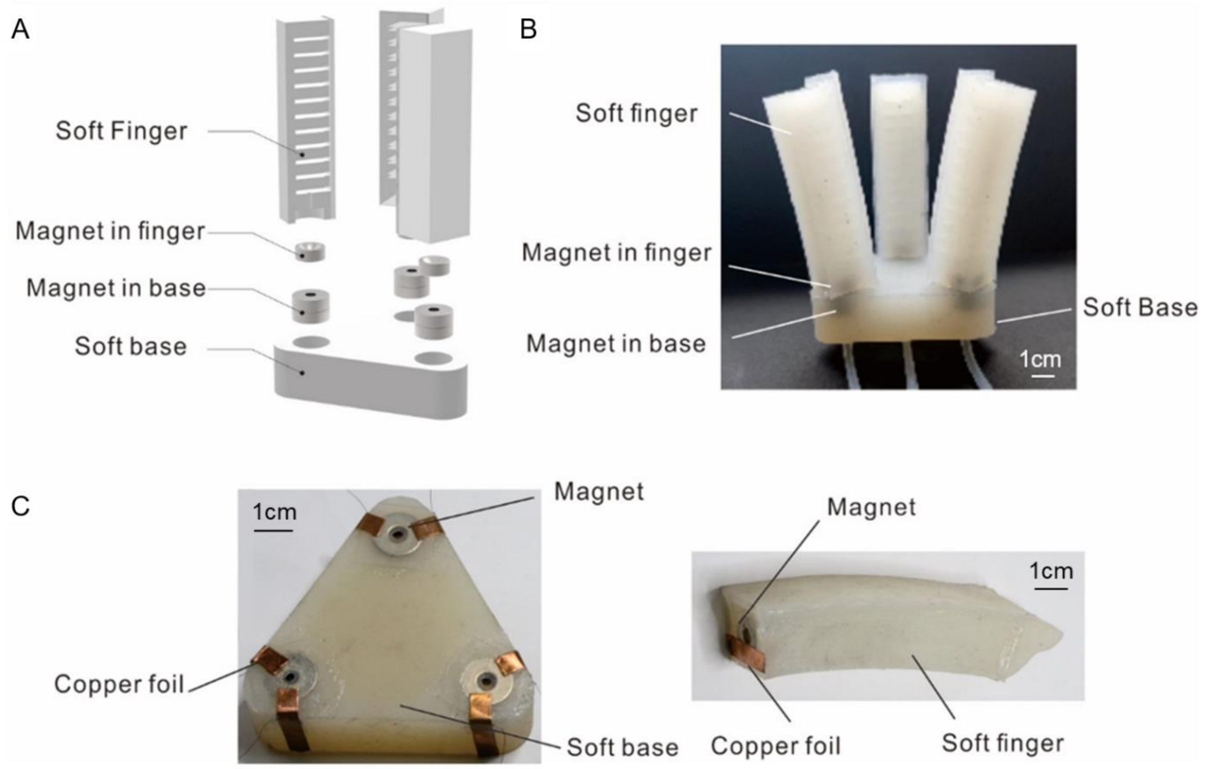

Figure S23. Design of modular pneumatic soft gripper based on magnetic connection. Panel (A) shows an explosion display of the structural design of the soft gripper. Panel (B) shows the side view of the modular pneumatic soft gripper. (C) Soft fingers can be assembled onto the soft base with magnetic connections to connect the circuits for functional diagnosis.

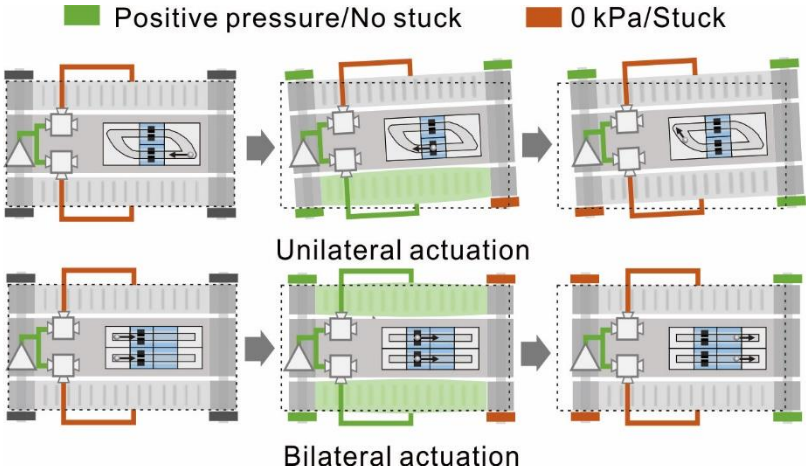

Figure S24. Schematic of working principle of different locomotion controlled by reconfiguration of fundamental modules. Through the periodic actuation of the two bending actuators, the soft car can twist its body in programmed order to move forward in different trajectories. During the moving process, the four wheels can be selectively stuck, transferring the deformation of the soft car into its body movement.

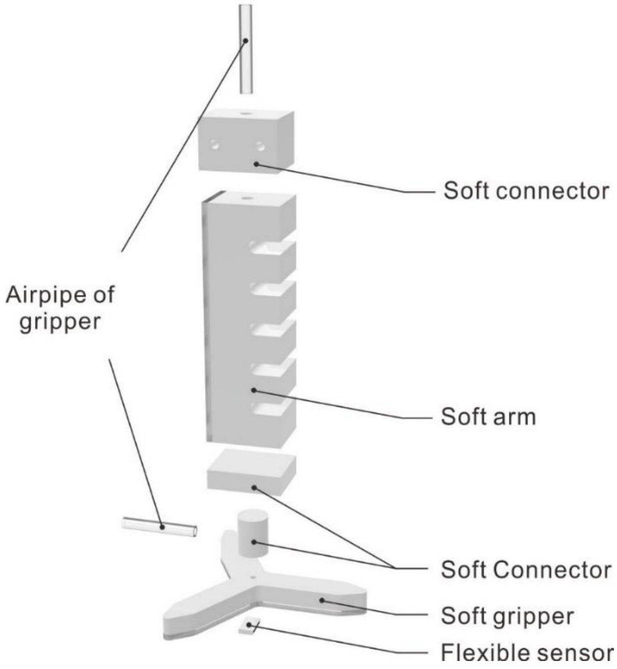

Figure S25. Structural design and actuation mechanism of the soft arm and gripper. The soft arm and gripper are consisted of soft materials with bending and gripping deformation by the supplied pressure via air pumps. The state of solenoid valves is controlled by contacting behavior between MLMD and electrodes of SRC. All the valves exhibit two states, either let the air pressure go through soft materials for actuation or to the atmosphere for relaxation. Therefore, the grasping and sorting behavior can be achieved automatically under the cyclic motion of MLMD.

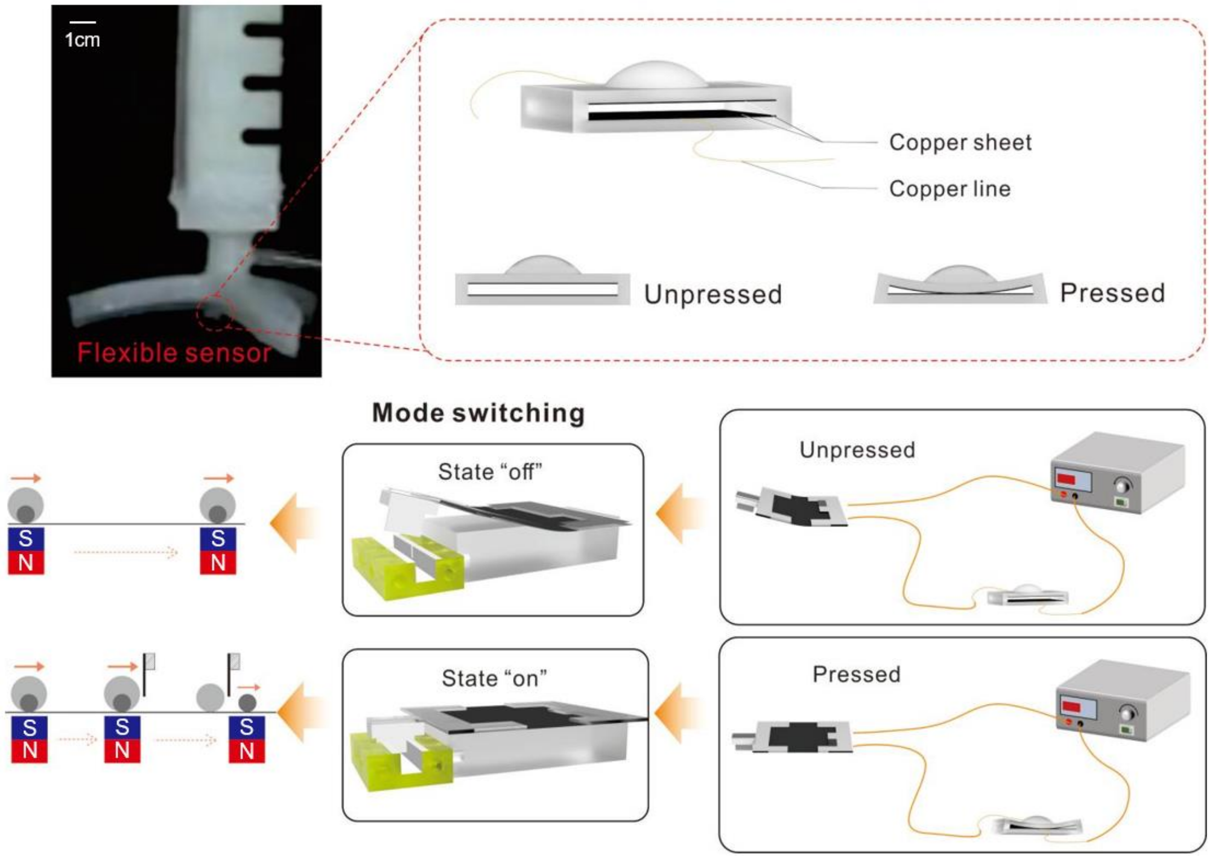

Figure S26. A flexible sensor for information storage. A flexible sensor integrated into the middle of the gripper is applied for contacting information storage to the SRC when the gripper interacts with the unstructured objects. Once the gripper reaches the targeted object, the lower surface will be pressed contacting with the upper surface. Noting that the distance between upper and lower surface is designed to be 0.8 mm. Therefore, a slight force acting on the lower surface will cause significant deformation due to the flexible material property. As the conducting behavior is activated, the DEA-based soft gate will be triggered by the high voltage source to implement "on" and "off" states to activate the MLMD splitting characteristics. As a consequence, the program switching mode is triggered for the sequential program to achieve sorting behavior.

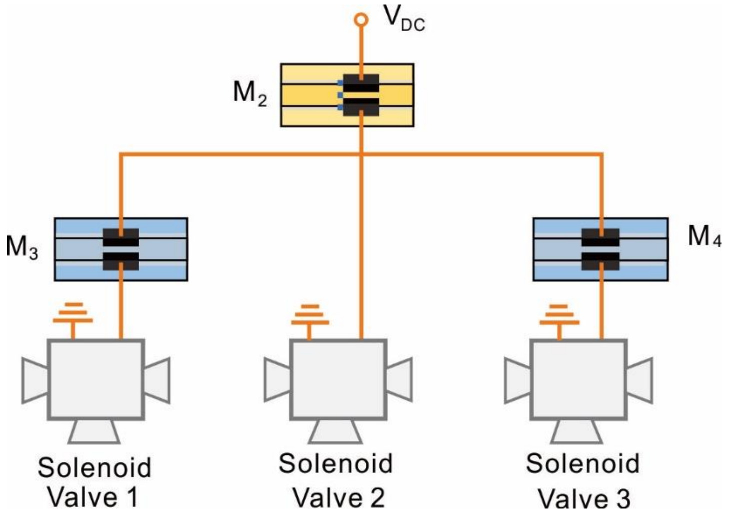

Figure S27. Program design with reconfigurable modules for self-adaptive control. The programming module (yellow) is applied for MLMD splitting behavior. Noting that the only if the electrode of yellow module is connected, the M3 and M4 will be conducted when the MLMD is contacting the electrodes. Therefore, the yellow module can not only store the information to trigger splitting behavior but also maintain the initial program and switch to another program. Therefore, the soft robot can automatically search the objects and sort them to the targeted place.

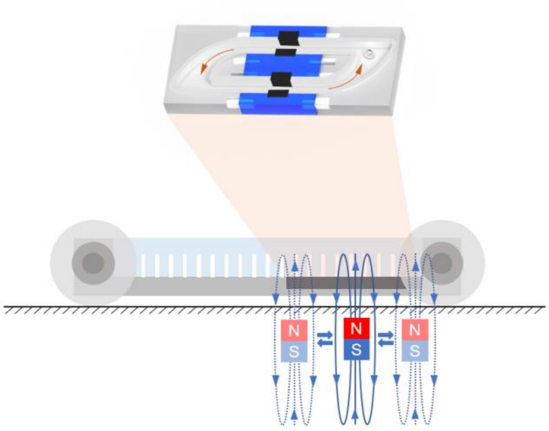

Figure S28. Schematic of a manual magnetic actuation method. A small permanent magnet was manually controlled to move periodically in a straight line at the bottom of the soft machines in our experiments to control the motion of MLMDs.

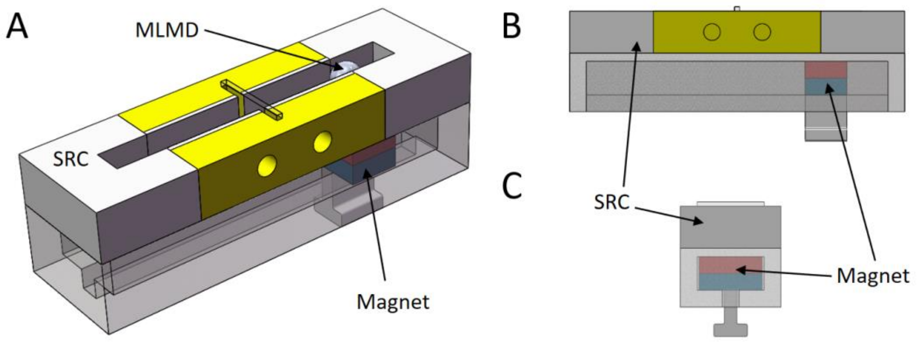

Figure S29. A SRC-based soft manual control switch. The SRCs can be integrated with a soft magnet slide switch to enable manual switching for soft systems, where (A)-(C) are the views of the soft manual control switch from different angles.

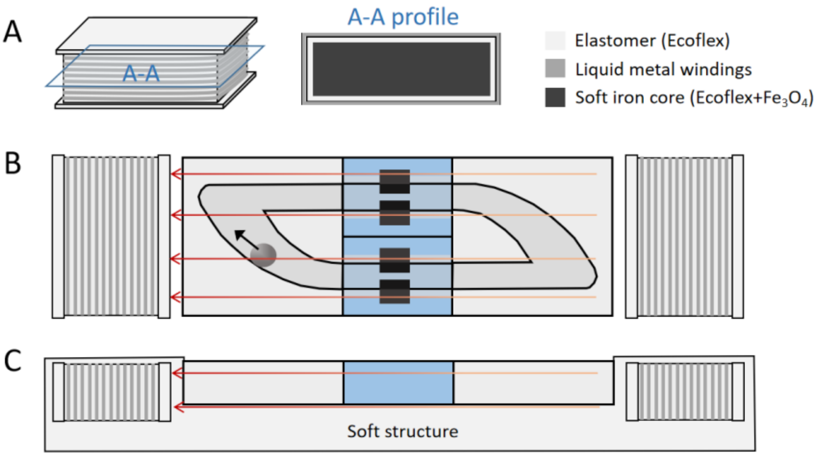

Figure S30. Schematic of a magnetic actuation method based on soft electromagnets to achieve fully onboard SRC systems without external input magnetic signals. (A) Schematic of a feasible structural scheme for a soft electromagnet. (B)-(C) Soft electromagnets can be arranged at both ends of the SRC and embedded into the soft structure. The MLMD's cycle can be controlled by the alternating activation of soft electromagnets.

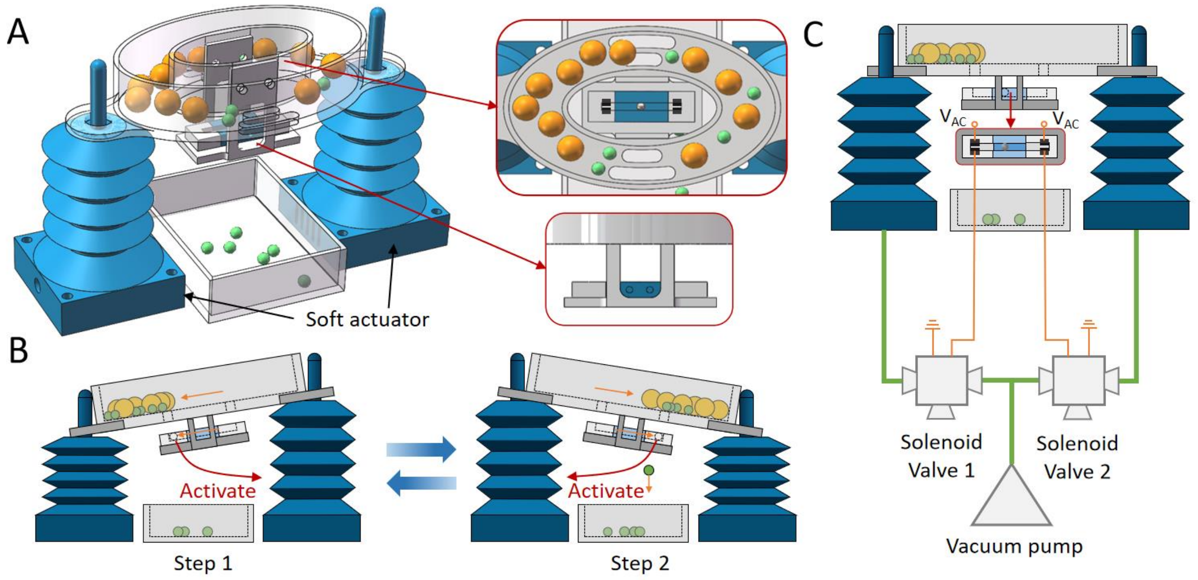

Figure S31. Schematic of a self-cyclic SRC-based screening oscillator under gravity.. (A) 3D diagram of the screening oscillator. (B) Cyclic schematic of the screening oscillators. (C) Airpath and circuit connection diagram of the screening oscillators.

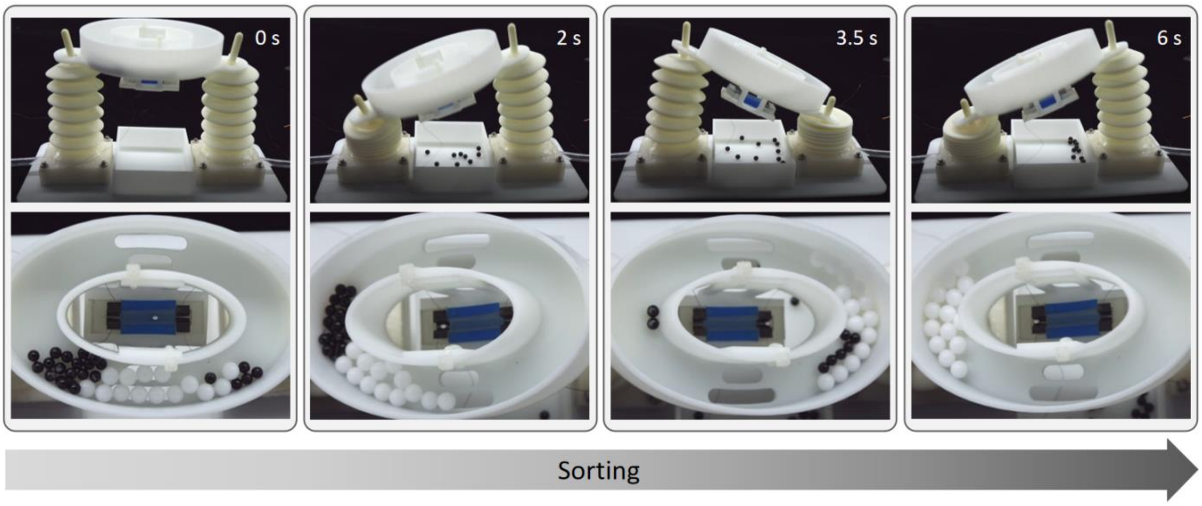

Figure S32. The screening oscillator can automatically achieve the screening of large and small spheres within 6 s by self-cyclic oscillation. The soft actuators were 3D printed with thermoplastic polyurethanes (TPU) material, while the rest of the parts were 3D printed by a light-curing 3D printer (UNION 3D Lite600) with WEILAI 8000 resin.

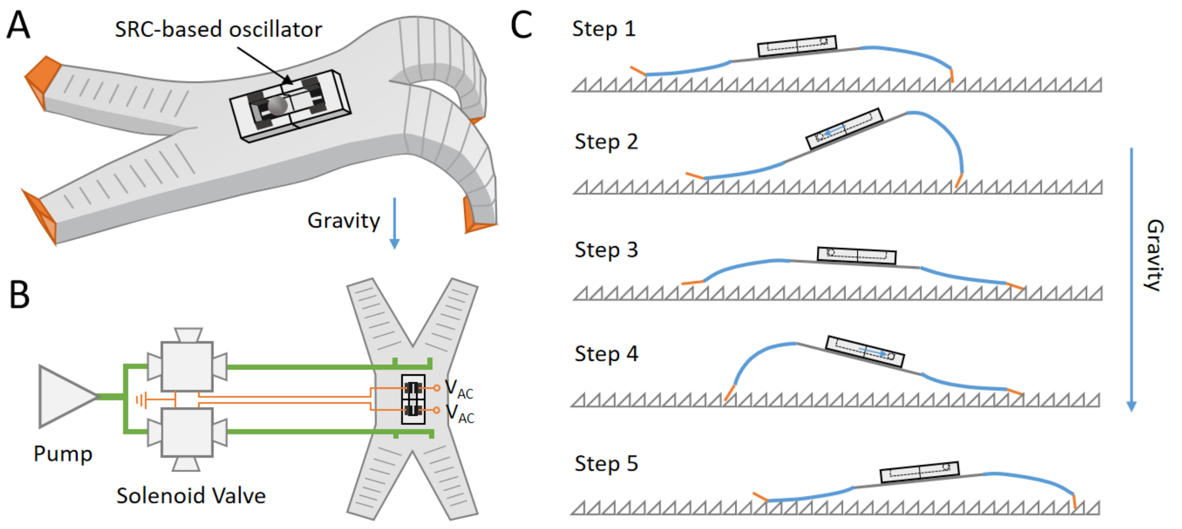

Figure S33. Schematic of the automatic crawling of a quadruped soft robot controlled by an SRC-based gravity-driven oscillator. (A) Schematic of the soft quadruped robot integrated with an SRC-based oscillator. (B) Airpath and circuit connection diagram of the soft quadruped robot. (C) Schematic of the self-cycling crawling gait of the soft quadruped robot.

References

[1] S. W. Kwok, S. A. Morin, B. Mosadegh, J. So, R. F. Shepherd, R. V. Martinez, B. Smith, F. C. Simeone, A. A. Stokes, G. M. Whitesides, *Adv. Funct. Mater.* **2014**, 24, 2180.

[2] L. Jin, A. E. Forte, K. Bertoldi, *Adv. Sci*. **2021**, 8, 2101941.

[3] L. C. van Laake, J. de Vries, S. M. Kani, J. T.B. Overvelde, *Matter*, **2022**, 5, 2898.

[4] W. K. Lee, D. J. Preston, M. P. Nemitz, A. Nagarkar, A. K. MacKeith, B. Gorissen, N. Vasios, V. Sanchez, K. Bertoldi, L. Mahadevan, G. M. Whitesides, *Sci Robot*, **2022**, 7, 63.

[5] P. Rothemund, A. Ainla, L. Belding, D. J. Preston, S. Kurihara, Z, Suo, G. M. Whitesides, *Sci Robot*, **2018**, 3,16.

[6] C. J. Decker, H. J. Jiang, M. P. Nemitz, S. E. Root, A. Rajappan, J. T. Alvarez, J. Tracz, L. Wille, D. J. Preston, G. M. Whitesides, *Proc. Natl. Acad. Sci*, **2022**, 119, 40.

[7] M. Garrad, G. Soter, A. T. Conn, H. Hauser, J. Rossiter, *Sci Robot*, **2019**, 4, 33.

[8] D. Li, T. Liu, J. Ye, L. Sheng, J. Liu, *Adv. Intell. Syst*, **2021**, 3, 2000246.

[9] B. H. Kim, K. Li, J.-T. Kim, Y. Park, H. Jang, X. Wang, Z. Xie, S. M. Won, H.-J. Yoon, G. Lee, W. J. Jang, K. H. Lee, T. S. Chung, Y. H. Jung, S. Y. Heo, Y. Lee, J. Kim, T. Cai, Y. Kim, P. Prasopsukh, Y. Yu, X. Yu, R. Avila, H. Luan, H. Song, F. Zhu, Y. Zhao, L. Chen, S. H. Han, J. Kim, S. J. Oh, H. Lee, C. H. Lee, Y. Huang, L. P. Chamorro, Y. Zhang, J. A. Rogers, *Nature*. **2021**, 597, 503.

[10] W. Tang, Y. Lin, C. Zhang, Y. Liang, J. Wang, W. Wang, C. Ji, M. Zhou, H. Yang, J.

Zou, *Sci. Adv*. **2023**, 7, eabf8080.
[11] J. Liang, Y. Wu, J. K. Yim, H. Chen, Z. Miao, H. Liu, Y. Liu, Y. Liu, D. Wang, W. Qiu, Z. Shao, M. Zhang, X. Wang, J. Zhong, L. Lin, *Sci. Robot*. **2021**, 6, eabe7906.
[12] W. Tang, C. Zhang, Y. Zhong, P. Zhu, Y. Hu, Z. Jiao, X. Wei, G. Lu, J. Wang, Y. Liang, Y. Lin, W. Wang, H. Yang, J. Zou, *Nat. Commun*. **2021**, 12, 2247.
[13] G. Mao, D. Schiller, D. Danninger, B. Hailegnaw, F. Hartmann, T. Stockinger, M. Drack, N. Arnold, M. Kaltenbrunner, *Nat. Commun*. **2022**, 13, 4456.
